# Alkylglycerol monooxygenase represses prostanoid biosynthesis in a sex-dependent manner

**DOI:** 10.1186/s13578-025-01419-5

**Published:** 2025-06-05

**Authors:** Zhigang Rao, Katharina Lackner, Ilaria Dorigatti, Natascha Brigo, Denise Kummer, Minh Bui Hoang, Christa Pfeifhofer-Obermair, Günter Weiss, Ernst R. Werner, Andreas Koeberle, Katrin Watschinger

**Affiliations:** 1 Michael Popp Institute and Center for Molecular Biosciences Innsbruck (CMBI), University of Innsbruck, Innsbruck, Austria; 2 Institute of Human Genetics, Medical University of Innsbruck, Innsbruck, Austria; 3 Institute of Molecular Biochemistry, Biocenter, Medical University of Innsbruck, Innsbruck, Austria; 4 Department of Internal Medicine II, Medical University of Innsbruck, Innsbruck, Austria; 5 Institute of Pharmaceutical Sciences and Excellence Field BioHealth, University of Graz, Graz, Austria

**Keywords:** AGMO, Ether lipid, Lipidomics, Lipid mediator, Polyunsaturated fatty acid

## Abstract

**Background:**

Ether lipids are important constituents of biological membranes and harbor fatty alcohols attached via ether linkages to the *sn*-1 position of the glycerol backbone. Depending on the nature of the ether bond, they are subdivided into 1-*O*-alkyl (plasmanyl) and 1-*O*-alk-1′-enyl (plasmenyl) subclasses. They often contain polyunsaturated fatty acids at the *sn-2* position, implicating them in cellular signaling and inflammatory processes including lipid mediator biosynthesis. Lipid mediators are produced by immune and non-immune cells, have diverse homeostatic and immunoregulatory functions and, together with other factors, orchestrate the initiation and resolution of inflammation. To date, alkylglycerol monooxygenase is the only known enzyme capable of cleaving alkylglycerols, one of two ether lipid subclasses. However, the exact role of alkylglycerol monooxygenase and that of its substrates in lipid mediator biosynthesis remains unclear.

**Results:**

Using a knockout mouse model, we demonstrate a sex- and cell type-dependent role of alkylglycerol monooxygenase in limiting prostanoid formation without affecting polyunsaturated fatty acid release, as revealed by metabololipidomics profiling of lipid mediators using ultra-performance liquid chromatography‒tandem mass spectrometry. This female-specific effect is driven by the suppression of prostaglandin G/H synthase 2 transcription, as deficiency in alkylglycerol monooxygenase significantly elevated prostaglandin G/H synthase 2 gene expression in female bone marrow-derived macrophages of the M1 phenotype. Furthermore, this regulatory role of alkylglycerol monooxygenase extends to visceral white adipose tissue, where elevated prostaglandin G/H synthase 2 expression and enhanced prostaglandin E_2_ production were observed in female samples following alkylglycerol monooxygenase knockout.

**Conclusion:**

Our results expand the immunomodulatory functions of ether lipid metabolism and highlight the sex- and cell type-dependent role of alkylglycerol monooxygenase in controlling lipid mediator production and maintaining tissue homeostasis.

**Supplementary Information:**

The online version contains supplementary material available at 10.1186/s13578-025-01419-5.

## Background

Ether lipids represent a class of glycerol-based lipids distinguished by the presence of an ether linkage at the *sn*-1 position, in contrast to the more prevalent ester-linked analogs. These lipids are further classified into two subclasses: 1-*O*-alkyl (plasmanyl) and 1-*O*-alk-1′-enyl (plasmenyl) ether lipids, the latter commonly referred to as plasmalogens containing a vinyl ether double bond at the *sn*-1 position. Both subclasses of ether lipids are found in glycerophospholipids, particularly in phosphatidylcholines (PC) and phosphatidylethanolamines (PE) [1]. Ether lipid biosynthesis starts in the peroxisomes under the action of two enzymes, glyceronephosphate *O*-acyltransferase (GNPAT, EC 2.3.1.42) and alkylglycerone phosphate synthase (AGPS, EC. 2.5.1.26). The rate-limiting enzymes fatty acyl-CoA reductases 1/2 (FAR1/2, EC 1.2.1.84) reduce fatty acyl-CoAs to provide the fatty alcohol needed by AGPS to introduce the ether bond at the *sn*-1 position of glycerol [2]. Final ether lipid maturation, remodelling and degradation takes place in the endoplasmic reticulum, where plasmanyl ether lipids can either be catabolized by alkylglycerol monooxygenase (AGMO, EC 1.14.16.5) [3] or further processed to plasmalogens by the enzyme plasmanylethanolamine desaturase (PEDS1, EC 1.14.19.77) [4].

So far, AGMO is the only known enzyme able to cleave alkylglycerols and lysoalkylglycerophospholipids (plasmanyl ether lipids) into the corresponding glycerol derivatives and a fatty aldehyde [5]. Following the initial description of the enzymatic reaction in 1964 [6], it took nearly five decades to identify the genetic sequence encoding for AGMO. In 2010, *Tmem195* was identified as the coding gene for AGMO through a combination of bioinformatic analyses and *in vitr*o expression studies in cell lines [3]. Studies on AGMO in murine bone marrow-derived macrophages (BMDMs) demonstrated a downregulation of both AGMO activity and gene expression upon treatment with lipopolysaccharide (LPS) and other pro-inflammatory stimuli, which induce M1 macrophage polarization. Additionally, *Agmo* knockdown in RAW264.7 macrophages was shown to perturb the cellular lipidome, influencing not only ether lipids but also other lipid classes [7]. Genetic association studies in humans have suggested a potential involvement of AGMO in various diseases, including relapses of visceral leishmaniasis [8], microcephaly [9, 10], autism [11], and congenital heart disease [12, 13]. Recently, our laboratory developed an *Agmo* knockout mouse model, which exhibits no apparent phenotype under normal, unchallenged conditions [14].

Alkylglycerols, the substrates of AGMO, have been proposed to play critical roles in cellular signaling [15]. For example, they modulate the activity of different ion channels and receptors, including toll-like receptor 4, peroxisomal proliferator-activating receptors, and G-protein-coupled receptors, partly as direct ligands [15]. The best studied alkylglycerols are platelet-activating factors (PAF, 1-*O*-alkyl-2-*O*-acetyl-*sn*-glycero-3-phosphocholine), which have pro-inflammatory activities, partly due to the induction of cyclooxygenase (COX)-2 (prostaglandin G/H synthase 2, PTGS2, EC 1.14.99.1) expression and prostanoid production [16–18], but also regulate homeostatic processes, e.g. related to neuronal function and reproduction [19]. PAF is synthesized in various cell types, including platelets, myeloid leukocytes (such as basophils, macrophages and monocytes), and endothelial cells, through either the *de novo* or remodeling pathway [20]. *De novo* PAF biosynthesis transfers phosphocholine to 1-*O*-alkyl-2-acetyl-*sn*-glycerols and is primarily involved in maintaining intracellular PAF levels. The remodeling pathway, which is induced by inflammatory stimuli, consists of two sequential steps: first, the acyl group at the *sn-*2 position of plasmanyl phosphatidylcholine (abundant in macrophages and other immune cells [21]) is cleaved by cytosolic phospholipase A_2_ (cPLA_2_, EC 3.1.1.4) to form lyso-PAF (also referred to as lysophosphatidylcholine). Second, lyso-PAF is acetylated at the *sn*-2 position by lyso-PAF acetyltransferases LPCAT2/LPLAT9 (EC 2.3.1.67) and potentially LPCAT1/LPLAT8 (EC 2.3.1.67) [22, 23]. Notably, AGMO has been shown to reduce pro-inflammatory PAF levels in transfected human HEK293 embryonic kidney cells [24]. The opposite effect was not evident after down-regulation of endogenous AGMO in murine RAW264.7 macrophages [7].

Alkylglycerols have also been associated with activating macrophages, promoting phagocytosis and enhancing the humoral immune response [25, 26]. Ether lipids often carry polyunsaturated fatty acids (PUFA) at their *sn-*2 position, which are preferentially cleaved by cPLA_2_ [27]. As such PUFA reservoirs they can influence cellular processes, including the biosynthesis of lipid mediators with pro-inflammatory, immunomodulatory, and homeostatic functions [28] as well as ferroptosis susceptibility [22].

Lipid mediators are synthesized from PUFA via (di)oxygenating pathways, categorized by the initial key enzymes: COXs, lipoxygenases (LOXs), and cytochrome P450 monooxygenases [29]. They play an essential role in tissue homeostasis and immunoregulation and contribute to the induction and resolution of inflammation [30]. For example, COX isoenzymes produce prostaglandin (PG) H_2_, the common precursors of PGs, thromboxanes (TXs) and prostacyclins [31]. While the constitutively expressed COX-1 (PTGS1, EC 1.14.99.1) primarily controls basal physiological functions including gastric mucosal protection and platelet aggregation, the inducible COX-2 (PTGS2) mediates the excessive formation of prostanoids during inflammation, in addition to possessing homeostatic functions [32].

In this study, we investigated the role of AGMO in lipid mediator biosynthesis in M1- and M2-like BMDMs and responsive mouse tissues, both under healthy conditions and during infection. Our study reveals cell type-specific and sex-dependent activities of AGMO in limiting the production of COX-derived prostanoids through restricting *Ptgs2* transcription in pro-inflammatory M1 BMDMs and visceral white adipose tissue (vWAT), the latter restricted to non-infectious conditions. These results enhance our current understanding of the physiological roles of AGMO, highlighting the broader biological significance of this enzyme and its ether lipid substrates.

## Materials and methods

### Chemicals

Most chemicals were purchased from Sigma-Aldrich (Schnelldorf, Germany), Roth (Karlsruhe, Germany), Thermo Fisher Scientific (Waltham, USA) and Serva (Heidelberg, Germany). Chemicals purchased by other companies are mentioned in the text.

### Knockout mouse line

*Agmo*-deficient mice (official line name: *Agmo*^tm1a(EUCOMM)Wtsi^) were generated from embryonic stem cells (clone EPD0354_2_F05, EuMMCR, Munich, Germany) and were maintained on C57BL/6 J background (for details see [14]). Mice were housed in individually ventilated cages with nesting material, in a 12 h/12 h light/dark cycle with standard chow (Ssniff Spezialdiäten GmbH, Soest, Germany; complete feed for rats and mice V1534-300, autoclaved) and water *ad libitum*. Animal breeding was approved by the Austrian Federal Ministry of Education, Science and Research (BMWFW-66.011/0094-WF/V/3b/2016). Genomic DNA of *Agmo*-deficient mice was extracted from ear notches using the Monarch^®^ Genomic DNA Purification Kit (New England Biolabs, Frankfurt am Main, Germany) and genotyping was performed by allele counting via qPCR, using SsoFast EvaGreen Supermix (Bio-Rad Laboratories Inc., Hercules, USA) and primers for *Agmo*-lacZ (fw 5’-TCTGTATGAACGGTCTGGTC-3’, rv 5’-TATTCGCTGGTCACTTCGAT-3) and reference gene *Eef2* (fw 5’-AGGCCTGTGTAATATAGCTGCG-3’, rv 5’-CTCTGTGTAGTTTGTAGCTCTGTCT-3’) (for details on the procedure see [14]).

### Isolation and culture conditions of bone marrow derived macrophages (BMDMs)

Mouse femur and tibia were isolated after cervical dislocation and were stored in PBS pH 7.4 or DMEM/F12 supplemented with 1% (v/v) penicillin/streptomycin (P/S) at 4 °C, not longer than 24 h until further processing. Under a laminar flow residual leg muscles were removed with sterile scissors or a scalpel and the ends of the bones were removed. The opened bones were placed into a 0.5 mL Eppendorf tube (femur and tibia of 1 leg in 1 tube) pierced with a sterile needle at the bottom to separate the bone marrow from the bones and this punctured 0.5 mL tube was then placed into a 1.5 mL Eppendorf tube for centrifugation at 21,100 × g for 3 s. The resulting cell pellet in the 1.5 mL Eppendorf tube containing the bone marrow was resuspended in 1 × PBS + 1% P/S and filtered through a 70 μm cell strainer using additional 10 mL PBS. The cell suspension was centrifuged at 230 × g for 5 min, the supernatant removed and incubated for 5 min with 10 mL of red blood cell (RBC) lysis buffer (154.4 mM ammonium chloride, 10 mM potassium bicarbonate, 193.5 µM EDTA dissolved in aqua destillata (a.d.) and filtered sterile). Subsequently, the suspension was centrifuged at 150 x g for 10 min, washed with PBS and centrifuged again before the cell pellet was resuspended in macrophage growth medium containing DMEM/F12 supplemented with 10% FCS (Gibco™), 1% P/S, 1% L-glutamine and 50 ng/mL M-CSF (PeproTech, East Windsor, UK) for macrophage proliferation. The cell suspension was split into 15 non-treated 100 mm^2^ cell culture dishes in a volume of 10 mL and at day 5 additional 5 mL of complete medium (containing FCS, P/S, L-glutamine and M-CSF) were supplemented. Cells were cultivated in macrophage growth medium as M0 macrophages for 6 days in a humidified atmosphere at 37 °C with 5% CO_2_.

### Harvesting of BMDMs and stimulation to M0, M1 and M2 macrophages

Macrophages were harvested by removal of the growth medium and addition of 5 mL ice-cold 1 × PBS/5 mM EDTA. After 3 min of incubation the solution was pipetted up and down on the dish to dislodge the cells which were counted with trypan blue in a Burker chamber. The cells were centrifuged and 2 × 10^6^/mL cells were plated in macrophage stimulation medium consisting of Gibco™ Optimem, 0.2% BSA, 1% P/S, 1% L-glutamine (if not already included in the medium) in cell culture treated 6-well plates. After letting cells adhere for 2 h, stimulants for M1 polarization (100 ng/mL LPS, 10 ng/mL IFN-γ, both from PeproTech) or M2 polarization (20 ng/mL IL-4, PeproTech) were added. After 24 h incubation, stimulants were added again. M0 macrophages were kept in macrophage stimulation medium without stimulants. After a total incubation time of 48 h cells were harvested for gene expression, AGMO activity assay and lipid mediator analysis.

### Isolation of PBMC from human blood and polarization of human macrophages

Human peripheral blood mononuclear cells (PBMC) were isolated from leukocyte reduction system chamber (LRSC) filters, which were provided by the Central Institute for Blood Transfusion and Immunological Department of Tirol Kliniken GmbH (Austria) with the informed consent of the volunteers. Only healthy blood donors between 18 and 65 years of age without medication for chronic diseases, fever, or deficiency symptoms and after physical examinations by trained medical personnel were included in the study. In brief, cell concentrates from the LRSC filters were diluted in pre-warmed acid-citrate-dextrose buffer containing 0.97% citric-acid-monohydrate, 2.25% dextrose, 2.2% trisodium citrate dihydrate and PBS. Immune cells were isolated via density gradient centrifugation (400 × g, 20 min, RT) using Histopaque-1077. After lysis of erythrocytes using ice-cold water (2 mL) and two washing steps with PBS, PBMC were obtained [33].

PBMC were differentiated to M0 macrophages with 20 ng/mL M-CSF or GM-CSF (Hiss Diagnostics, Freiburg, Germany) for 6–7 days in macrophage medium containing RPMI 1640 supplemented with 10% FCS, 100 U/mL penicillin, and 100 µg/mL streptomycin. These macrophages were further polarized into M1 or M2 phenotypes within 48 h using 20 ng/mL IFN-γ (R&D Systems, Minneapolis, MN) and 100 ng/mL of LPS or 20 ng/mL IL-4 (Peprotech, Rocky Hill, US), respectively [34, 35].

### Determination of cell viability

Cell viability was measured with CellTiter-Blue^®^ Cell Viability Assay (Promega, Madison, USA) according to the manufacturer’s protocol. In brief, 200 µL of cell titer blue reagent were added per well (6-well containing 1 mL stimulation medium) and incubated for 1 h. A blank control was analyzed simultaneously containing solely 1 mL stimulation medium without cells. Afterwards, 100 µL of the incubated medium-cell titer blue mix was transferred as duplicates into a 96-well plate and fluorescence was measured (excitation: 560, emission: 590).

### Quantification of protein content

Polarized BMDMs (M0, M1 and M2 phenotypes) were lysed in ice-cold lysis buffer containing Tris-HCl (20 mM, pH 7.4), NaCl (150 mM), EDTA (2 mM), NaF (5 mM), phenylmethanesulphonyl fluoride (1 mM), leupeptin (1 mg/mL), soybean trypsin inhibitor (60 µg/mL), sodium vanadate (1 mM), and sodium pyrophosphate (1 mM). After lysates had been sonicated (3 × 3 s, on ice) and centrifuged (21,100 × g, 5 min, 4 °C), cell supernatants were collected and their protein concentration determined by DC-protein assay kit (Bio-Rad Laboratories GmbH, Munich, Germany).

### AGMO activity assay

BMDMs (M0, M1 and M2 phenotypes) were harvested by scraping of the cells, centrifugation at 16,000 × g, snap freezing the cell pellets in liquid nitrogen and storing them at -80 °C. The frozen cell pellet was resuspended in 20–40 µL lysis buffer consisting of a.d., 0.5% CHAPS, 1 mM dithioerythritol (DTE) and 1 × protease inhibitor (GE Healthcare, Chicago, USA) depending on the amount of harvested cells and protein concentration was measured by the Bradford assay with serum albumin as standard to normalize the enzymatic activity. AGMO activity assay was measured as described in [36] with the following minor modifications: Fatty aldehyde dehydrogenase was added in its recombinant form to the assay mixture and a final concentration of 1 mM DTE in the assay was supplemented [37]. We always analyzed samples and controls in parallel to exclude artifacts by an intrinsic day-to-day variability of the assay.

### RNA isolation and gene expression analysis by quantitative PCR

BMDMs (M0, M1 and M2 phenotypes) were harvested by scraping of the cells, centrifugation at 16,000 × g, snap freezing the cell pellets in liquid nitrogen and storing them at -80°C. Total RNA from snap frozen cell pellets of M0, M1 and M2 macrophages was prepared using the Monarch^®^ Total RNA Miniprep Kit (New England Biolabs, Frankfurt am Main, Germany) according to the manufacturer’s protocol. Transcription into complementary DNA was performed using the M-MLV reverse transcriptase (RNase H Minus, Point Mutant; Promega, Mannheim, Germany) and random hexamer primers (Microsynth, Balgach, Switzerland). For quantitative PCR (qPCR), the TaqMan assay technology using Luna^®^ Universal Probe qPCR Master Mix (New England Biolabs) and the Mx3005P qPCR system (Agilent, Vienna, Austria) were used. TaqMan probes were labeled with fluorescein (FAM) (5′) and tetramethylrhodamine (TAMRA) (3′). Primer sequences were as follows (all purchased from Microsynth): *18S*: 5’ CCATTCGAACGTCTGCCCTAT 3’ (sense), 5’ TCACCCGTGGTCACCATG 3’ (antisense), 5’ ACTTTCGATGGTAGTCGCCGTGCCT 3’ (probe); *Agmo* (Exon 3_4): 5’ CTTTCTTAGGAGTTGACTTTGGCTACT 3’ (sense), 5’ TGTGCTGCCCAGAAAATATTAATC 3’ (antisense), 5’ CTGGTTCCACCGCATGGCTCATG 3’ (probe); *iNos*: 5’ TCCCTCCTGATCTTGTGTTGG 3’ (sense), 5’ CAACCCGAGCTCCTGGAAC 3’ (antisense), 5’ TGACCATGGAGCATCCCAAGTACGAGT 3’ (probe); *Arg1*: 5’ TGGTGGCAGAGGTCCAGAA 3’ (sense), 5’ TGGCCAGAGATGCTTCCAA 3’ (antisense), 5’ ACTGTGGTCTCCACCCAGCACCACA 3’ (probe); *Ptgs1*: 5’ GAAGTACTCATGCGCCTGGTACT 3’ (sense), 5’ GTAGTCATGCGCTGAGTTGTAGGT 3’ (antisense), 5’ ACAGTGCGGTCCAACCTTATCCCCA 3’ (probe); *Ptgs2*: 5’ GCTCAGCCAGGCAGCAAA 3’ (sense), 5’ TCAAATCCTGTGCTCATACATTCC 3’ (antisense), 5’ CCTTGCTGTTCCAATCCATGTC 3’ (probe); *Pla2g4*: 5’ TTTGAGTTCATTTTGGATCCTAATCA 3’ (sense), 5’ TGTAGCTGTGCCTAGGGTTTCA 3’ (antisense), 5’ CCATGACGTAGTTGGCATCCATCAGTGT 3’ (probe); *Alox5*: 5’ AATCTTCGTCAAAATCAGCAACAC 3’ (sense), 5’ TGGTAGCCAAACATGAGGTCTTC 3’ (antisense), 5’ TCTGAGCGAGTCAAGAACCACTGGCA 3’ (probe); *Alox5ap*: 5’ GCCTTTGAGCGGGTCTACAC 3’ (sense), 5’ AGTCCAGAGTACCACAAGGAAAGTG 3’ (antisense), 5’ CCAACCAGAACTGCGTAGATGCGTACC 3’ (probe); *Il-1b*: 5’ ACCTGTCCTGTGTAATGAAAGACG 3’ (sense), 5’ TGGGTATTGCTTGGGATCCA 3’ (antisense), 5’ CACACCCACCCTGCAGCTGGAGA 3’ (probe); *Tnfa*: 5’ GGCCTCCCTCTCATCAGTTCT 3’ (sense), 5’ AGCTGCTCCTCCACTTGGTG 3’ (antisense), 5’ TGGCCCAGACCCTCACACTCACAA 3’ (probe); *Tgfb1*: 5’ GCTCTTGTGACAGCAAAGATAACAA 3’ (sense), 5’ GGTCGCCCCGACGTTT 3’ (antisense), 5’ CACGTGGAAATCAACGGGATCAGCC 3’ (probe);

### Sample preparation and metabololipidomics analysis of lipid mediators

After 48 h cytokine stimulation, polarized BMDMs (M0, M1 and M2 phenotypes) were stimulated with *Staphylococcus aureus*-conditioned medium (SACM, 1%, 3 h) or A23187 (2.5 µM, 15 min) for the indicated time. To prepare SACM, *Staphylococcus aureus* (6850) was grown in a brain heart infusion medium for 18 h at 37 °C under orbital shaking (160 × rpm). The bacteria suspension was then centrifuged (3,400 × g, 10 min, 20 °C), the supernatant was sterile filtered through a filter unit (0.22 μm; Millipore, Burlington, MA) and recovered as SACM [38]. Cell supernatants (in 1.5 mL PBS + 1 mM CaCl_2_) were collected for solid phase extraction of lipid mediators.

Murine fat tissues were collected after cervical dislocation and homogenized in 500 µL methanol using stainless-steel beads and two cycles of 2 min and 20 Hz in a Retsch MM400 homogenizer (Retsch, Haan, Germany). For sWAT, the protocol of the homogenizer was adjusted to three cycles of 2 min and 30 Hz.

Cell supernatants (1.5 mL) or tissue homogenates (150 µL) were mixed with ice-cold methanol (3 mL) containing the deuterated internal standards *d*_8_-5 S-HETE, *d*_4_-LTB_4_, *d*_5_-LXA_4_, *d*_5_-RvD2, *d*_4_-PGE_2_ (20 pg/µL, 10 µL each, Cayman Chemicals, Ann Arbor, MI) and *d*_8_-arachidonic acid (200 pg/µL, 10 µL, Cayman Chemicals). The sample mixtures were first kept at -20 °C for more than 2 h to allow protein precipitation, and then centrifuged (1200 × g, 10 min, 4 °C). Supernatants were transferred to new vials and mixed with 8 mL of acidified water (pH = 3.5) before loading onto Sep-Pak C18 6 cc Vac Cartridges (500 mg; Waters, Milford, MA) [39] pre-equilibrated with 6 mL methanol and 2 mL water. After several washing steps with water (6 mL) and *n*-hexane (6 mL), lipid mediators were eluted with methyl formate. The eluate was brought to dryness using a TurboVap system (TurboVap LV, Biotage, Uppsala, Sweden) under nitrogen flow at 30 ^o^C.

The dried lipid film was dissolved in methanol/water (50/50, v/v) and analyzed by ultra-performance liquid chromatography‒tandem mass spectrometry (UPLC-MS/MS) [40, 41]. The Exion LC system (Sciex, Darmstadt, Germany) was coupled to a QTRAP 6500+ mass spectrometer (Sciex), which was equipped with a Turbo V source and electrospray ionization source. Lipid mediators were separated on a reversed phase column (ACQUITY UPLC^®^ BEH C18; 1.7 μm; 2.1 mm × 100 mm; Waters) at a flow rate of 0.35 mL/min at 55 °C. The mobile phase was composed of A (10% methanol, 90% water, 0.01% acetic acid) and B (100% methanol + 0.01% acetic acid), which was ramped from 35.6% B to 84.4% B over 12.5 min, then to 97.8% B and maintained for another 5 min, and finally isocratically set to 35.6% B for 2.5 min. The QTRAP 6500+ mass spectrometer was operated in negative ionization mode using scheduled multiple reaction monitoring (detection window: 120 s). Optimized source MS parameters are shown in Table 1. Parameters for single lipid mediators including Declustering Potential, Entrance Potential, Collision Energy, and Collision Cell Exit Potential were optimized (Table 2).

**Table 1:** Optimized source MS parameters for lipid mediator analysis

parameters	
Curtain gas	40 psi
Collision gas	medium
Ion spray voltage	-4000 eV (negative mode)
Heated capillary temperature	500 °C
Sheath gas pressure	40 psi
Auxiliary gas	40 psi

Mass spectra were acquired using Analyst 1.7.1 (Sciex) and processed with Analyst 1.6.3 (Sciex), where the retention time was defined with the help of external lipid standards. The absolute amount of lipid mediators was calculated from integrated signal intensities that were (1) normalized using the deuterated internal standards, (2) corrected by external calibration (linear regression based on 11 dilutions), and (3) divided by the amount of protein in cell lysates or tissue weight.

**Table 2:** MRM transitions and MS parameters of single lipid mediators

diagnostic ion	Q1	Q3	Declustering Potential	Entrance Potential	Collision Energy	Collision Cell Exit Potential
d_8_-5 S-HETE	327.3	116.1	-80	-10	-17	-10
d_4_-LTB_4_	339.3	197.2	-80	-10	-22	-13
d_4_-PGE_2_	355.3	193.2	-80	-10	-25	-16
d_5_-LXA_4_	356.3	115.2	-80	-10	-19	-14
d_5_-RvD2	380.3	141.2	-80	-10	-23	-14
d_8_-AA	311.3	267.1	-100	-10	-16	-18
17-HDHA	343.2	245.1	-80	-10	-17	-14
14-HDHA	343.2	205.1	-80	-10	-17	-14
7-HDHA	343.2	141.1	-80	-10	-18	-15
4-HDHA	343.2	101.1	-80	-10	-17	-15
17-HDPA	345.2	247.1	-80	-10	-17	-14
14-HDPA	345.2	207.1	-80	-10	-17	-14
7-HDPA	345.2	143.1	-80	-10	-18	-15
18-HEPE	317.2	259.1	-80	-10	-16	-23
15-HEPE	317.2	219.1	-80	-10	-18	-12
12-HEPE	317.2	179.1	-80	-10	-19	-12
11-HEPE	317.2	167.1	-80	-10	-19	-12
5-HEPE	317.2	115.1	-80	-10	-18	-12
15-HETE	319.2	219.1	-80	-10	-19	-12
12-HETE	319.2	179.1	-80	-10	-21	-12
11-HETE	319.2	167.1	-80	-10	-21	-12
5-HETE	319.2	115.1	-80	-10	-21	-12
5S,6R-diHETE	335.2	115.1	-80	-10	-20	-13
PGD_2_	351.3	189.1	-120	-10	-20	-13
PGE_2_	351.2	271	-120	-10	-20	-13
15-ketoPGE_2_	349.2	235.1	-115	-6	-19	-11
PGF_2_α	353.3	193.1	-80	-10	-34	-11
TXB_2_	369.3	169.1	-80	-10	-22	-15
PGE_1_	353.2	317.4	-90	-10	-18	-15
LTB_4_ isomers	335.2	195.1	-80	-10	-22	-13
LTB_4_	335.2	195.1	-80	-10	-22	-13
5,15-diHETE	335.2	201	-50	-10	-30	-13
AA	303.3	259.1	-100	-10	-16	-18
EPA	301.3	257.1	-100	-10	-16	-18
DHA	327.3	283.1	-100	-10	-16	-18
DPA	329.3	285.1	-100	-10	-16	-18

### Quantitative analysis of (lyso-) platelet-activating factor (PAF)

Cell pellets (stimulated without or with Ca^2+^-ionophore A23187 or SACM) were resuspended in PBS (150 µL) and mixed with ice-cold methanol (365 µL) containing deuterated internal standards (*d*_4_-PAF-C16 and *d*_4_-lyso-PAF-C16, 200 pg/µL, 10 µL each). Chloroform (twice 187.5 µL) and saline (0.9% sodium chloride, 187.5 µL) were sequentially added and the samples thoroughly mixed (twice, 30 s each) after each step. Following centrifugation (3,000 × g, 5 min, 4 °C), the chloroform layer was collected and brought to dryness using a SpeedVac vacuum concentrator [42].

The lipid film was dissolved in methanol for UPLC-MS/MS analysis using a QTRAP 6500+ mass spectrometer (Sciex), equipped with a Turbo V source for electrospray ionization and coupled to an Exion LC system (Sciex). Lipids were separated on a reversed phase column (1.7 μm, 2.1 × 50 mm, Waters) at a flow rate of 0.8 mL/min at 45 °C. The mobile phase was composed of A (100% acetonitrile, 0.07% formic acid) and B (10% acetonitrile, 90% water, 0.07% formic acid). Isocratic elution with 30% A was maintained for 2 min and then ramped to 70% A within 5 min, which was kept isocratic for 2 min before being switched to 30% A for another 2 min [43]. The QTRAP 6500+ was operated in negative ionization mode using scheduled multiple reaction monitoring. Optimized source MS parameters are listed in (Table 3). The MS parameters for the individual metabolites are shown in (Table 4) [43].

**Table 3:** Optimized source MS parameters for PAF and lyso-PAF analysis

parameters	
Curtain gas	40 psi
Collision gas	medium
Ion spray voltage	-4500 eV (negative mode)
Heated capillary temperature	450 °C
Sheath gas pressure	50 psi
Auxiliary gas	40 psi

Mass spectra were acquired with Analyst 1.7.1 (Sciex) and processed with Analyst 1.6.3 (Sciex). Retention times were defined using external standards. To calculate the absolute amount of PAF-C16 and lyso-PAF-C16, signal intensities were corrected by external calibration (linear regression based on 11 concentrations) and normalized to the deuterated internal standard and the amount of cellular protein.

**Table 4:** MRM transitions and MS parameters of PAF, d_4_-PAF, d_4_-lyso-PAF and lyso-PAF

diagnostic ion	Q1	Q3	Declustering Potential	Entrance Potential	Collision Energy	Collision Cell Exit Potential
d_4_-PAF-C16	572	59	-70	-10	-85	-10
d_4_-lyso-PAF-C16	530	530	-70	-10	-85	-10
PAF-C16	568	59	-70	-10	-5	-22
Lyso-PAF-C16	526	526	-70	-10	-5	-22

### *Salmonella* infection

The *Salmonella enterica* serovar Typhimurium (*S*.tm) ATCC14028 wildtype strain (ATCC, Manassas, Virginia, USA), which serves as the mouse model for *Salmonella Typhi*, was utilized for in vivo infection experiments following the previously described protocol [44]. Briefly, 50 µL of an overnight culture, prepared with 10 µL of *S*.tm and 10 mL of Lysogeny Broth (LB) medium, was diluted in 10 mL of fresh LB medium and incubated with shaking at 37 °C until reaching an OD600 of 0.5. The viable *S*.tm were then counted using the Casy counting system (OMNI Life Science, Bremen, Germany). A dose of 1,000 viable *S*.tm in 200 µL of PBS was administered intraperitoneally (i.p.) to 8–12 week-old male or female *Agmo* knockout mice and wildtype controls, respectively. Uninfected mice were injected with 200 µL PBS (Lonza, Basel, Switzerland). After 72 h of infection, the mice were sacrificed by cervical dislocation and fat tissues (vWAT, sWAT, BAT) were collected for analysis. The animal experiments were approved by the Austrian Federal Ministry of Science and Research (GZ-2022.0.344-283).

### Statistics and data presentation

GraphPad Prism (version 9.0.1 or 10.2.2) was used for all statistical data analyses and data presentation. All experiments were repeated at least three times and n numbers are indicated in the respective figures. Data were visualized as heat-maps, bars and whisker plots with individual data points and data are presented as mean ± s.e.m. Outliers were excluded by Grubbs’ test. For analysis of statistical significance, ordinary two-way ANOVA plus Tukey or Sidak *post hoc* tests, or two-tailed unpaired *t*-test to determine differences between two groups or two-way ANOVA to compare multiple groups was used. *p* < 0.05 was considered significant in each test.

## Results

### AGMO activity and expression of murine BMDMs

**Fig. 1:**
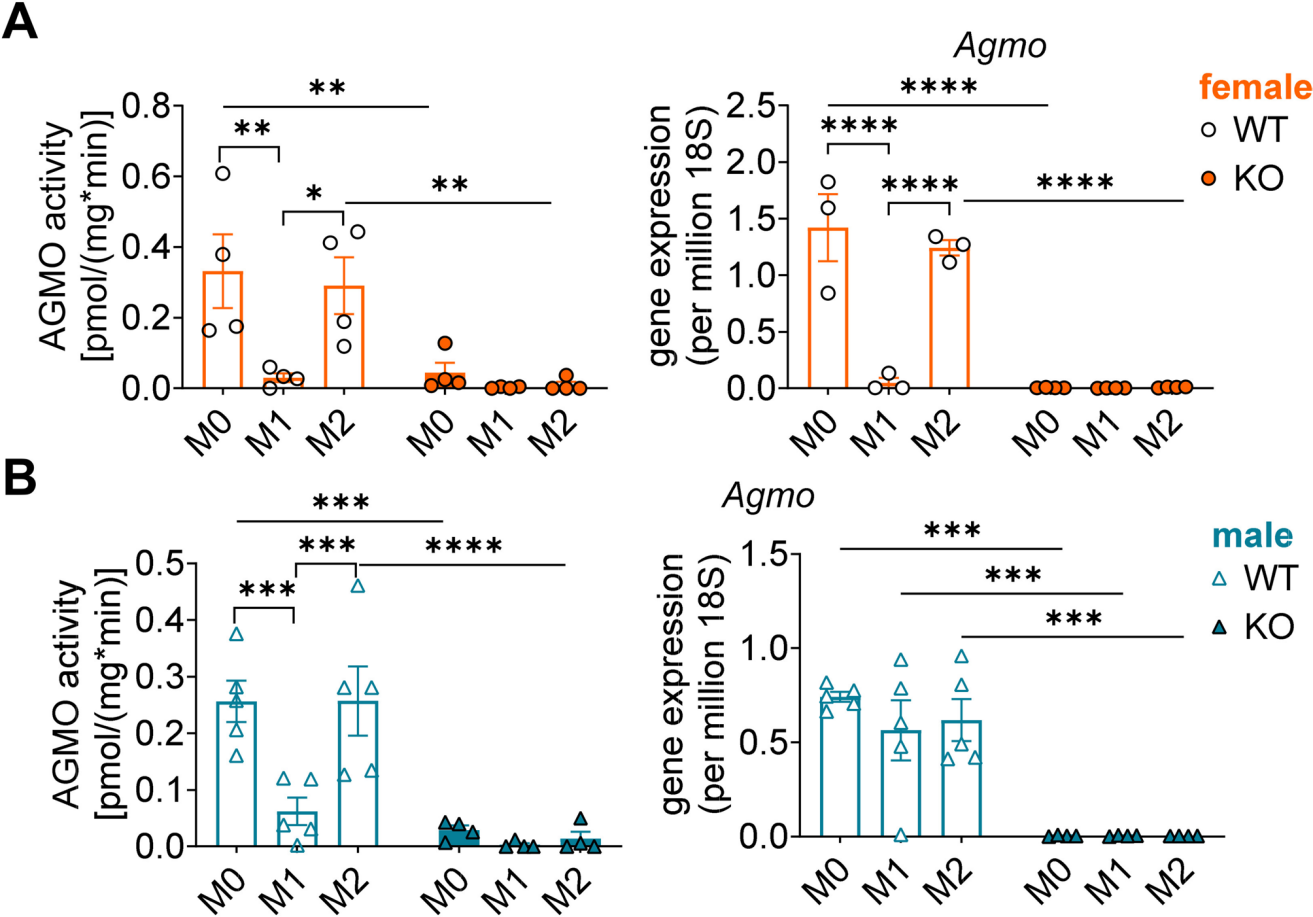
AGMO activity and expression in polarized murine BMDMs. Murine BMDMs isolated from wildtype (WT, open circles/triangles) or *Agmo* knockout (KO, filled circles/triangles) mice (female, orange; male, blue) were polarized to the M0, M1 or M2 phenotypes (for a schematic overview of polarization refer to Fig. S1A). AGMO activity (left panels) and *Agmo* gene expression (right panels) in M0, M1 or M2 from (**A**) female and (**B**) male mice are shown. Data are shown as mean ± s.e.m; *n* = 3–5. ****p < 0.0001, ***p < 0.001, **p < 0.01, *p < 0.05; ordinary two-way ANOVA plus Tukey *post hoc* tests

Ether lipids harbor PUFA such as arachidonic acid (AA) at their *sn*-2 position, thus serving as a reservoir for the biosynthesis of lipid mediators that play a role in pro-inflammatory and immunomodulatory processes [22, 28]. To investigate how the ether lipid-cleaving enzyme AGMO impacts the lipid mediator profile, bone marrow cells were isolated from femur and tibia of wildtype (WT) and *Agmo* knockout (KO) female and male mice and differentiated to BMDMs with macrophage colony stimulating factor (M-CSF). After 6 days they were either kept unpolarized (M0) or polarized for 48 h into M1 and M2 phenotypes with interferon-γ and LPS or interleukin-4, respectively (Fig. S1A). Successful polarization was verified by gene expression analysis of *iNos* and *Arg1*, established M1 and M2 markers, respectively (Fig. S1B, C). Both markers were higher in female than in male mice (female vs. male; *iNos*, 3.4-fold, *p* = 0.0041; *Arg1*, 2.7-fold, *p* = 0.1275; two-tailed unpaired *t*-test). AGMO activity and gene expression in M0, M1 and M2 WT macrophages were in line with previous findings [7], with unchanged levels between M0 and M2, while in M1 a strong downregulation could be detected in activity of male and female BMDMs and expression in female BMDMs, but unexpectedly not in males. The expression of *Agmo* was significantly higher in female M0 and M2 BMDMs, but markedly lower in female M1 BMDMs compared to the male counterparts (female vs. male; M0: *p* = 0.0214, M1: *p* = 0.0528, M2; *p* = 0.0072; two-tailed unpaired *t*-test; Fig. 1A, B). In BMDMs from KO mice, AGMO activity and expression were hardly detectable in all conditions, thus demonstrating successful depletion of AGMO in this cell type (Fig. 1A, B). While *Agmo* loss had no substantial impact on M1 BMDMs polarization, as *iNos* levels were comparable between KO and WT mice, the M2 marker *Arg1* was significantly elevated in M2 BMDMs from female KO mice and reduced in male KO mice (Fig. S1B, C). Cell viability and protein amount remained consistent across all BMDM phenotypes from both WT and KO mice, irrespective of sex, indicating that polarization did not interfere with these parameters (Fig. S1D, E).

### AGMO restricts COX product formation in M1 BMDMs in a sex-dependent manner

**Fig. 2:**
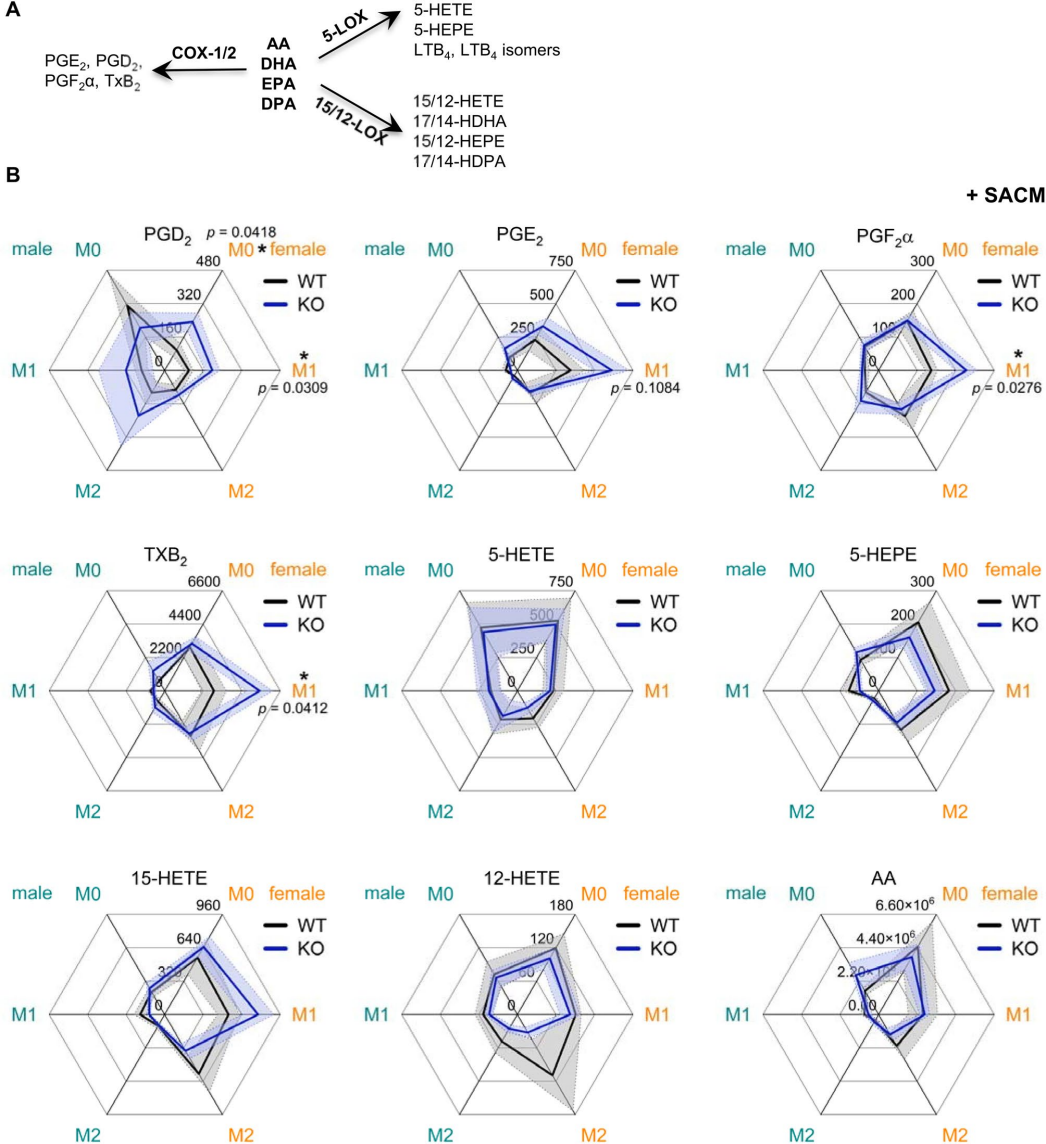
AGMO restricts COX product formation in M1 BMDMs in a sex-dependent manner. **A**) Simplified scheme of the biosynthetic pathway of lipid mediators. COX: cyclooxygenase, LOX: lipoxygenase. (**B**) Murine BMDMs isolated from wildtype (WT) or *Agmo*-deficient (KO) mice (female, orange; male, blue) were polarized to the M0, M1 or M2 phenotypes, and then stimulated with *S. aureus*-conditioned medium (SACM, 1% v/v, 3 h). Lipid mediators were extracted and analyzed by UPLC-MS/MS. The levels of exemplary lipid mediator species (pg/0.2 mg protein) are shown in the radar-plots. Data are shown as mean ± s.e.m, the mean values were connected by solid lines (WT, black; KO, blue) and the s.e.m values were connected by dashed lines (WT, black; KO, blue). *n* = 4–5. *p < 0.05; two tailed unpaired *t*-tests

Under resting conditions, BMDMs produce moderate eicosanoid levels (Table. S1, S4), which increase upon exogenous stimulation [45]. To boost lipid mediator biosynthesis in BMDMs, we made use of *Staphylococcus aureus*-conditioned medium (SACM) [38] and the Ca^2+^-ionophore A23187 [38, 46]. SACM strongly elevated the formation of most lipid mediators (Fig. S2, Table. S2 and S5), including COX, 5-, 12-, and 15-LOX products (as shown in scheme Fig. 2A), while the ability of A23187 to increase lipid mediator biosynthesis was substantially lower (Fig. S2, Table. S3 and S6). Therefore, further studies on these metabolites focused on SACM-stimulated BMDMs.

BMDMs from female mice produced lipid mediators at higher levels (total lipid mediator amount in M0, M1 and M2: 6808.1, 4186.3 and 3184.0 ng/0.2 mg protein) as compared to those from male mice (total lipid mediator amount in M0, M1 and M2: 2585.0, 1361.9 and 668.0 ng/0.2 mg protein; Fig. 2B, Table. S2 and S5). The deletion of *Agmo* further elevated the biosynthesis of COX products, preferentially in the M1 phenotype - a sex-specific effect not observed in male BMDMs (Fig. S3). Surprisingly, this is the phenotype (M1) where low *Agmo* levels were observed. In particular, the levels of SACM-stimulated COX-derived PGD_2_, PGE_2_, PGF_2_α, and TXB_2_ were approximately two times higher in M1 BMDMs from female KO mice; compared to those from WT mice (Fig. 2B, Table. S2). Effects on M0 and M2 BMDMs were less pronounced, except for an increase in PGD_2_ formation in activated M0 BMDMs from female KO mice (Fig. 2B). *Agmo* deletion also influenced the levels of other lipid mediators in a sex-dependent manner, though effects were not consistent throughout lipid mediator subclasses or subjected to high variance, lacking statistical significance (Fig. 2B).

A similar but weaker trend towards elevated COX-derived product formation was evident in resting female M1 BMDMs without exogenous stimulation (Fig. S4, Table. S1). The AGMO-dependent formation of COX-derived products observed in female M1 BMDMs was not conserved in male BMDMs, where *Agmo* deficiency failed to induce significant changes in these lipid mediators (Fig. 2B and Fig. S4). The formation of lipid mediators from other classes, including 5-, 12-, and 15-LOX products as well as free fatty acids (i.e., AA), was barely changed across BMDM phenotypes following *Agmo* KO, regardless of sex (Fig. 2B and Fig. S4).

Taken together, our data reveals clear sex-dependent effects of AGMO on modulating the biosynthetic capacity of BMDMs to produce specific lipid mediator classes, with AGMO limiting COX-derived product formation specifically in female M1 BMDMs.

### AGMO limits *Ptgs2* expression in female M1 BMDMs

**Fig. 3:**
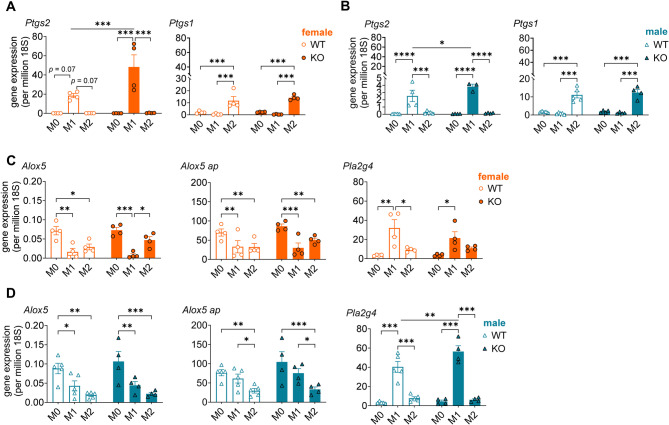
AGMO limits *Ptgs2* expression in M1 BMDMs in female mice. Murine BMDMs isolated from wildtype (WT, open circles/triangles) or *Agmo* knockout (KO, filled circles/triangles) mice (female, orange; male, blue) were polarized to the M0, M1 or M2 phenotypes. The gene expression of *Ptgs2*, *Ptgs1*,* Alox5*,* Alox5ap*, and *Pla2g4* in M0, M1 or M2 from **A**, **C**) female and **B**, **D**) male mice is shown. Data are shown as mean ± s.e.m; *n* = 4–5. ****p < 0.0001, ***p < 0.001, **p < 0.01, *p < 0.05; ordinary two-way ANOVA plus Tukey *post hoc* tests

To elucidate the mechanisms through which *Agmo* deletion upregulates COX product formation in BMDMs, we investigated the mRNA expression of major enzymes in lipid mediator biosynthesis in resting BMDMs of *Agmo* KO and WT mice without SACM stimulation. Focus was placed on COX isoenzymes (*Ptgs1*, *Ptgs2*), LOXs (*Alox5*, *Alox12*, *Alox15*), 5-lipoxygenase-activating protein (*Flap*, *Alox5ap*), and cPLA_2_ (*Pla2g4*). Consistent with the increase in COX product formation, *Agmo* deletion upregulated the expression of *Ptgs2* but not *Ptgs1* in M1 BMDMs, an effect that was more prominent in female cells (KO vs. WT; 2.6-fold, *p* = 0.0007; Fig. 3A) than in male cells (KO vs. WT; 1.5-fold, *p* = 0.0203; Fig. 3B). *Agmo* deletion did not alter the expression of enzymes involved in 5-LOX product formation, *Alox5* and *Alox5ap*, or *Pla2g4*, which releases AA/20:4 from membrane phospholipids and, like *Ptgs2*, is upregulated during M1 polarization (Fig. 3C, D). The mRNA expression of enzymes involved in 15/12-LOX product formation (i.e. *Alox12* and *Alox15*) was below the detection limit in both KO and WT BMDMs. This (iso)enzyme expression pattern is in line with a functional role of AGMO preferentially in regulating COX product formation and suggests that the increased COX product formation in KO female BMDMs is driven by the induction of *Ptgs2*.

**Fig. 4:**
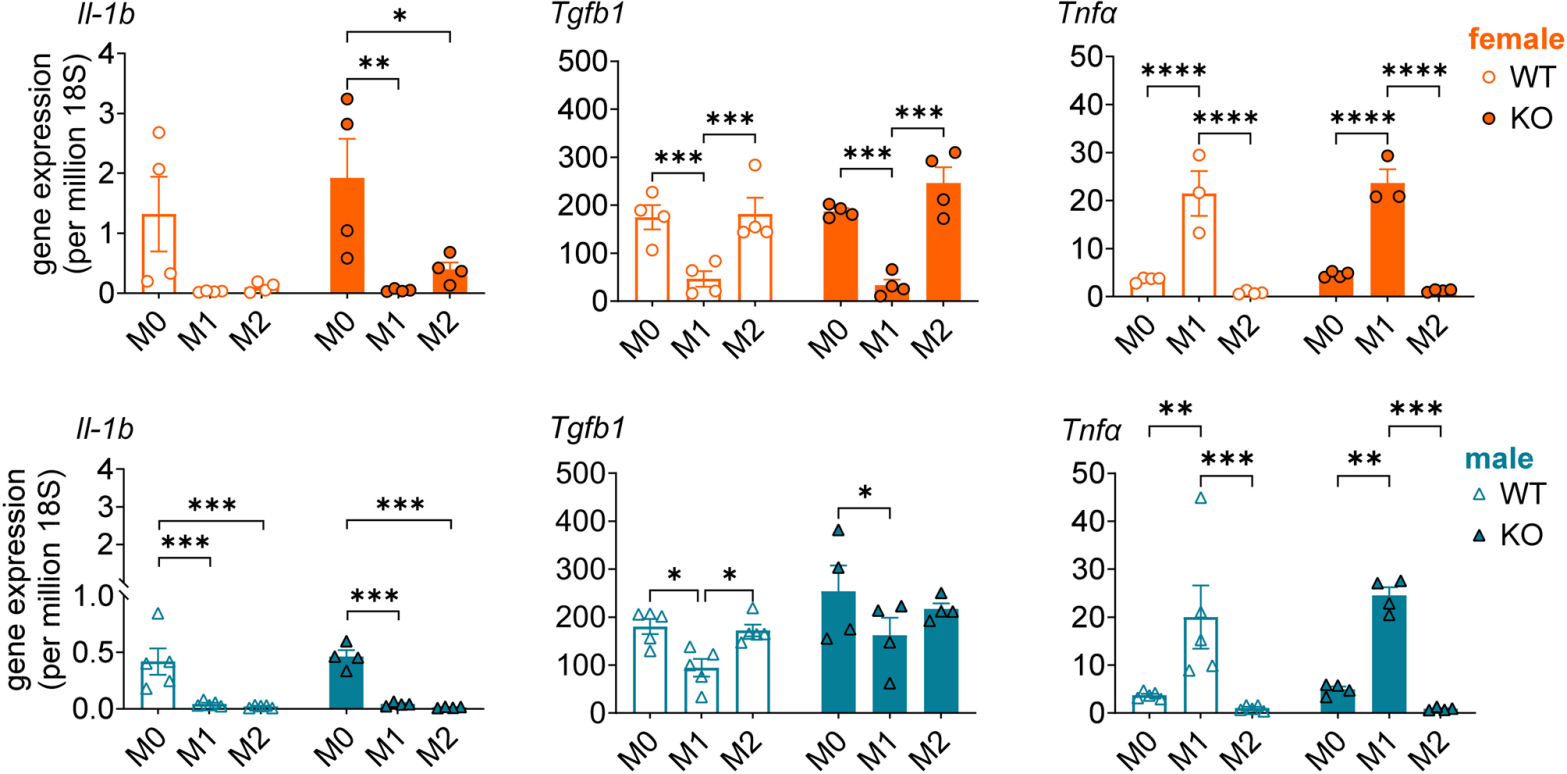
Agmo deficiency does not affect inflammatory cytokines in murine BMDMs. Murine BMDMs isolated from wildtype (WT, open circles/triangles) or *Agmo* knockout (KO, filled circles/triangles) mice (female, orange; male, blue) were polarized to the M0, M1 or M2 phenotypes. The gene expression of *Il-1b*, *Tgfb1* and *Tnfα* in M0, M1 or M2 from female and male mice is shown. Data are shown as mean ± s.e.m; *n* = 4–5. ****p < 0.0001, ***p < 0.001, **p < 0.01, *p < 0.05; ordinary two-way ANOVA plus Tukey *post hoc* tests

As PTGS2-derived prostanoids modulate the inflammatory response by regulating cytokine expression [47], we determined whether the effect of *Agmo* KO in M0, M1, and M2 BMDMs on the mRNA levels of pro-*Ptgs2* expression also influenced the levels of inflammatory and immunoregulatory cytokines (interleukin 1β (*Il-1b*), tumor necrosis factor α (*Tnfa*) and transforming growth factor-β1 (*Tgfb1*)). Apart from the expected regulation of M1 and M2 polarization [48], we could not detect major differences between KO and WT BMDMs, except for a 4-fold higher *Il-1b* gene expression in female *Agmo* KO M2 BMDMs, which was, however, not significant (Fig. 4).

AGMO has also been postulated to have an impact on the levels of platelet-activating factor (PAF), an ether phospholipid-derived mediator with pro-inflammatory function [24, 48]. To test this hypothesis, we analyzed PAF and lyso-PAF by UPLC-MS/MS in WT and KO BMDMs both under resting conditions and after immunogenic stimulation with SACM or induction of Ca^2+^-influx using the ionophore A23187. PAF and lyso-PAF levels remained unchanged irrespective of the presence or absence of AGMO (Fig. S5A, B), consistent with our previous findings [7]. Note that PAF levels were low in murine BMDMs, and both SACM and A23187 did not markedly increase them (Fig. S5A), in contrast to our control experiment using human macrophages, in which A23187 successfully elevated PAF levels (Fig. S5C).

Overall, the gene expression analysis strongly suggests that AGMO sex-dependently regulates COX-derived prostanoid levels mainly through *Ptgs2* transcription, which is specifically upregulated in KO female M1 BMDMs.

### AGMO modulates *Ptgs2* expression and COX product formation in adipose tissues

Next, we explored the role of AGMO in regulating *Ptgs2* transcription in selected tissues, i.e., liver, stomach, heart, kidney and three different types of fat tissue (vWAT; subcutaneous white adipose tissue, sWAT; brown adipose tissue, BAT). In these organs and tissues, PTGS2 shows substantial basal expression (https://www.proteinatlas.org/, https://www.ncbi.nlm.nih.gov/gene/19225), is induced by pro-inflammatory signaling [49, 50], and exhibits both pathological and homeostatic functions [51]. For example, it essentially contributes to prostanoid biosynthesis under inflammatory conditions, including those associated with liver cirrhosis [51, 52] and obesity-associated inflammation in adipose tissue [53]. PTGS2 also provides protection against non-alcoholic steatohepatitis, liver fibrosis [54], and supports gastric mucosal defense [55] and renal homeostasis [56].

**Fig. 5:**
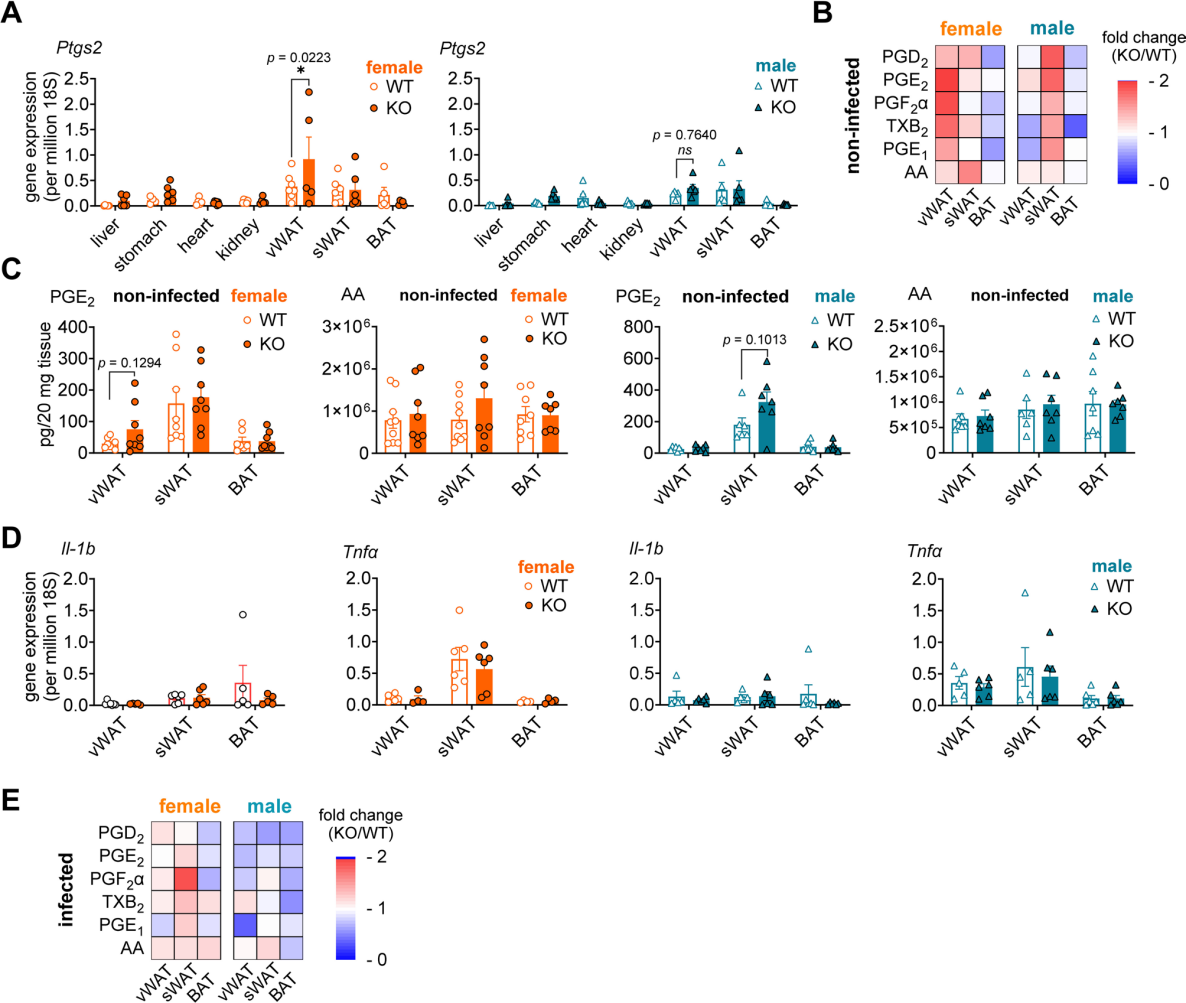
AGMO modulates *Ptgs2* expression and COX product formation in adipose tissues. **A**, **D**) Gene expression of **A**) *Ptgs2* and **D**) *Il-1b* and *Tnfα* in tissues isolated from wildtype (WT, open circles/triangles) or *Agmo* knockout (KO, filled circles/triangles) mice (female, orange; male, blue) (vWAT: visceral white adipose tissue, sWAT: subcutaneous white adipose tissue, BAT: brown adipose tissue). **B**, **C**, **E**) Female and male mice were **B**, **C**) non-infected or **E**) challenged with *Salmonella enterica* serovar Typhimurium (*S*.tm) ATCC14028 wildtype strain (infected) for 3 days, before adipose tissues (vWAT, sWAT and BAT) were collected and homogenized. Lipid mediators were extracted and analyzed by UPLC-MS/MS. **B**, **E**) The heatmaps show the changes in the levels of COX metabolites and arachidonic acid (AA) in the murine tissues. Color codes indicate the fold change of lipid mediator levels in tissues from KO against WT mice. **C**) Exemplary levels of PGE_2_ and AA. Data are shown as **A**, **C**, **D**) mean ± s.**e**.m or **B**, **E**) mean; **A**-**D**) *n* = 5–9, **E**) *n* = 3–5. *p < 0.05, ns: not significant; **A**) ordinary two-way ANOVA plus Sidak *post hoc* tests; **C**, **D**) two-tailed unpaired *t-*tests

First, we compared *Ptgs2* mRNA expression in respective tissues from WT and KO mice. Strongest differences were detected in vWAT of female mice, where *Ptgs2* mRNA expression in *Agmo* KO mice was 2.4-fold higher than in WT controls (Fig. 5A), with a similar trend in male vWAT. The differences in *Ptgs2* expression in the other tissues were less pronounced (Fig. 5A).

Further studies to determine the effect of AGMO on prostanoid profiles focused on adipose tissues. The deletion of *Agmo* was confirmed in all adipose tissues from KO mice on mRNA level (Fig. S6A). Consistent with the increase in *Ptgs2* expression in vWAT from female mice, *Agmo* deletion tended to increase the levels of PTGS2 products, including PGE_2_ (WT: 29.93 ± 5.82 pg/20 mg tissue vs. KO: 75.38 ± 27.59 pg/20 mg tissue) (Fig. 5B, C and Table. S7), an effect that was restricted to female mice. Interestingly, male KO mice showed a trend towards higher prostanoid levels in sWAT (Fig. 5B, C and Table. S7), independent of *Ptgs2* transcription. In contrast to BMDMs, *Agmo* deletion did not affect the expression of the pro-inflammatory cytokine *Il-1b* and *Tnfa* in adipose tissues (Fig. 5D).

To investigate whether *Agmo* deletion influences these parameters in adipose tissue under inflammatory conditions where *Ptgs2* is already upregulated [49], we infected mice with a *Salmonella enterica* serovar Typhimurium (*S*.tm) and analysed *Ptgs2* mRNA, prostanoid and cytokine levels. *Salmonella* infection in WT mice decreased *Agmo* expression in female vWAT, but not in other tissues from female mice or in any tissues from male mice (infected vs. non-infected; female vWAT, 0.15-fold, *p* = 0.0007; female sWAT, 0.55-fold, *p* = 0.3693; female BAT, 0.91-fold, *p* = 0.7874; male vWAT, 0.35-fold, *p* = 0.2764; male sWAT, 0.83-fold, *p* = 0.7671; male BAT, 2.01-fold, *p* = 0.4358; two-tailed unpaired *t*-test, Fig. S6A). In contrast to our findings in non-infected mice, *Agmo* deletion (confirmed at the mRNA level, Fig. S6A) did not induce *Ptgs2* expression in vWAT or other adipose tissues (Fig. S6B) and showed only mild effects on prostanoid levels, some with high variance (Fig. 5E and Fig. S6D). Note that *Ptgs2* is already highly expressed in adipose tissue of infected mice (Fig. S6B) and that the availability of the *Ptgs2* substrate AA is also substantially increased (Fig. S6C, D). Similar to our findings in non-infected mice and murine BMDMs, *Agmo* deletion had little effect on cytokine transcription in adipose tissue under infection, except for a clear trend towards higher *Il-1b* and *Tnfa* levels in female KO BAT samples (Fig. S6E).

In summary, our data reveal sex-dependent effects of AGMO in limiting *Ptgs2* expression and impeding COX product formation under both infectious (for M1 BMDMs) and homeostatic, non-infectious experimental settings (for M1 BMDMs and specific adipose tissues).

## Discussion

In this study, we uncover a sex-dependent role of AGMO in modulating COX-derived prostanoid formation, primarily through the upregulation of *Ptgs2* transcription in female M1 BMDMs and specific adipose tissues.

Although ether lipids have been implicated in various biological functions [20], the role of AGMO [3] still remains poorly understood. Given that ether lipids are enriched in PUFA [57], their release might serve as substrates for bioactive lipid mediators. Using an *Agmo* KO mouse model [14], we investigated how AGMO modulates lipid mediator biosynthesis in murine M0, M1 and M2 BMDMs, building on our previous findings of differential *Agmo* expression levels in these immune cell phenotypes [7]. Interestingly, the deletion of *Agmo* did not affect overall lipid mediator production by altering PUFA substrate availability, despite our previous data showing a clear increase in alkyl and alkenyl ether lipids under *Agmo*-deficient conditions [7]. Most probably, the unaltered free AA levels can be attributed to rapid turnover, where release is balanced by efficient incorporation into other (phospho)lipids [58].

Notably, *Agmo* KO selectively elevated the production of COX-derived prostanoids in a cell type-dependent manner. The rate-limiting step in prostanoid biosynthesis is the oxygenation of AA by COX, resulting in the formation of PGH_2_, an intermediate that is subsequently converted into prostanoids by specific synthases [31]. COX exists in two isoforms, COX-1 and COX-2, which are encoded by *Ptgs1* and *Ptgs2*, respectively. Our data indicate that the AGMO-mediated prostanoid formation is driven by *Ptgs2* expression, specifically in the M1 phenotype, with no significant contribution from *Ptgs1*. This cell type-specific effect may be linked to the differential expression of *Ptgs1* and *Ptgs2* isoforms in WT M0, M1 and M2 BMDMs. While both isoforms exhibit low basal levels in M0 BMDMs, *Ptgs2* is upregulated during M1 polarization in the presence of LPS and interferon-γ, and *Ptgs1* expression is elevated in IL4-induced M2 phenotypes. This pattern aligns with previous findings in BMDMs [59, 60] and human macrophages [59, 61]. Conversely, LPS has been reported to downregulate *Agmo* expression and activity in RAW264.7 macrophages [24] and adipose tissue macrophages [62], consistent with our observations in BMDMs. In LPS/interferon-γ-exposed M1 BMDMs, activity and expression levels of AGMO were lower compared to their M0 or M2 counterparts, and were nearly completely abolished in *Agmo* KO cells, where ether lipid levels are expected to further increase. Therefore, it is plausible to hypothesize that AGMO and/or its ether lipid substrates interfere with signaling components, which regulate inflammatory stress and are shared with the LPS signaling pathway that induces *Ptgs2* transcription.

Ether lipids, including plasmalogens and alkyl ether lipids, play crucial roles in cellular signaling, modulating inflammatory and immune responses [15]. Plasmalogens are predominantly recognized for their anti-inflammatory properties [63], whereas alkyl ether lipids exhibit both anti-inflammatory and pro-inflammatory activities, depending on their structural characteristics and biological context. The upregulation of *Ptgs2* through pro-inflammatory alkyl ether lipids, particularly alkylglycerols and alkyl glycerophospholipids, which are AGMO substrates, is a possible mechanism. One notable pro-inflammatory alkyl glycerophospholipid is PAF, with its precursor lyso-PAF serving as a substrate of AGMO [64]. Tokuoka et al. demonstrated that exogenous AGMO expression in HEK293 cells reduced cellular levels of both lyso-PAF and PAF, which aligns with the tetrahydrobiopterin-dependent degradation of lyso-PAF to glycerophosphocholine (GPC) described in RAW264.7 macrophages [24]. Further supporting a functional role for AGMO in PAF degradation, AGMO downregulation under LPS-induced pro-inflammatory conditions correlated with reduced GPC and elevated PAF and lyso-PAF levels [24]. In contrast, we did not observe significant changes in PAF or lyso-PAF levels in RAW264.7 macrophages following *Agmo* knockdown or overexpression [7] or in BMDMs (this study) upon *Agmo* KO. Notably, PAF levels in murine BMDMs were low, and neither PAF nor lyso-PAF levels responded to stimulation with SACM or the Ca^2+^-ionophore A23187. This contrasts with previous reports of A23187-induced PAF biosynthesis in human monocyte-derived macrophages and murine peritoneal macrophages [65, 66]. Thus, the potential involvement of AGMO in the regulation of PAF and lyso-PAF availability seems to be highly context- and cell type-dependent.

Less is known about alkylglycerols other than plasmalogens, PAF and lyso-PAF in immunoregulation. The interpretation of findings is further complicated by the fact that alkylglycerols are the direct plasmalogen precursors. There is also conflicting evidence regarding the direction of the effect. For example, alkylglycerols have been shown to exert anti-inflammatory actions in a human crossover study [67]. However, in murine lymphocytes, they were reported to promote the production of pro-inflammatory Th1 cytokines, such as IFN-γ and TNFα [68], the latter not confirmed in our study. More recently, Zou et al. identified PUFA-containing ether phospholipids as key drivers of ferroptosis [69], a process in which increased *Ptgs2* expression serves as a biomarker [70]. In fact, the oxidized alkyl glycerophospholipid 1-*O*-hexadecyl-2-azelaoyl-*sn*-glycero-3-phosphocholine has been reported to induce COX-2 expression and PGE_2_ secretion in human monocytes [71], supporting the hypothesis that alkylglycerols, PUFA-containing ether lipids and their oxidized derivatives modulate inflammatory pathways regulating *Ptgs2* expression and prostanoid production. Further evidence for the connection between ether lipids and prostanoid biosynthesis is substantiated by studies demonstrating that silencing AGPS, one of the two enzymes responsible for initiating ether lipid biosynthesis, reduced PGE_2_ production in human glioma and hepatic carcinoma cell lines [72].

Sex-specific effects have been observed in both human and murine models, where it has been demonstrated that sex hormones, as well as non-hormonal sex-associated genetic factors, influence the immune response to infections [73, 74]. In our experimental settings, we observed sex-specific effects on *Agmo* expression across all BMDM phenotypes: female M0 and M2 BMDMs exhibited higher *Agmo* expression, whereas female M1 BMDMs showed lower expression, compared to male counterparts. This pattern correlated with significantly higher *Ptgs2* expression and prostanoid formation in female M1 BMDMs, which was further elevated upon *Agmo* KO specifically in this phenotype. Note that AGMO activity is comparably low in both male and female M1 BMDMs, despite *Agmo* expression being slightly higher in males. Furthermore, AGMO deletion does not cause a further significant decrease in activity. These findings may explain why there are no major sex- or AGMO-dependent differences in inflammatory responses (i.e. cytokine expression) under inflammatory conditions. All the more intriguing is the fact that basal COX product formation is regulated by AGMO in M1 macrophages in a sex-dependent manner. Similar trends were observed in vWAT, where basal *Ptgs2* expression was higher in non-infected female tissues and *Agmo* KO increased *Ptgs2* expression and PGE_2_ production more than in males. PTGS2 and its derived prostanoids play dual and context-dependent roles in adipose tissue biology. While they are crucial for maintaining adipose tissue homeostasis, their effects appear paradoxical in obesity-related contexts. For example, PTGS2/prostanoids exhibit protective functions by reducing fat accumulation [75] and alleviating adipose tissue dysfunction [76] in obesity or high-fat mice models. Conversely, they also exert pro-inflammatory effects, promoting adipose tissue inflammation [53, 77].

Interestingly, these sex-dependent differences by *Agmo* KO were attenuated under *Salmonella* infection, likely due to the associated dominant upregulation of *Ptgs2* in both WT and *Agmo* KO tissues. Sex differences in PG biosynthesis have been shown to be diverse. Thus, Pace et al. reported that male human and murine neutrophils are more efficient at producing PGE_2_, correlating with higher COX-2 expression [78]. In contrast, Shen et al. identified higher PGD_2_ synthase expression in female human dorsal root ganglia neurons compared to males [79]. Given the context-dependent and sex-specific nature of prostanoid biosynthesis, our findings underscore the importance of considering sex differences in immunoregulation and further contribute to our understanding of this complex regulation.

## Conclusions

Taken together, our study reveals a sex-dependent role of AGMO in modulating *Ptgs2* expression and COX-derived prostanoid biosynthesis in M1 BMDMs and adipose tissues. This female-specific effect is independent of PAF and appears to be mediated by as yet unknown AGMO substrates or products that control the transcription of *Ptgs2*. While our study identified sex-specific upregulation of Ptgs2 in vWAT of AGMO-KO female mice, we acknowledge that further investigation of adipose tissue metabolism (e.g., differentiation, fat deposition) and systemic effects (e.g., body weight, inflammation, and food intake) in chow versus high fat diet would provide deeper mechanistic insights. These aspects, though beyond the current scope, represent important directions for future research.

## Electronic supplementary material

Below is the link to the electronic supplementary material.

Supplementary Material 1

## Data Availability

The mass spectrometric data for lipid mediators generated in this study have been deposited in the Metabolomics Workbench (10.21228/M8J53Q; Project ID: PR002430; Study ID: ST003876).

## Abbreviations

a.d.: Aqua destillata
AA: Arachidonic acid
AGMO: Alkylglycerol monooxygenase
AGPS: Alkylglycerone phosphate synthase
BAT: Brown adipose tissue
BMDM: Bone marrow-derived macrophage
COX: Cyclooxygenase
cPLA_2_: Cytosolic phospholipase A_2_
FAR: Fatty acyl-CoA reductases
GNPAT: Glycerone-phosphate O-acyltransferase
KO: Knockout
LOX: Lipoxygenase
LPCAT: Lyso-PAF acetyltransferase
LPS: Lipopolysaccharide
LRSC: Leukocyte reduction system chamber
M-CSF: Macrophage colony stimulating factor
PAF: Platelet-activating factor
PBMC: Peripheral blood mononuclear cells
PC: Phosphatidylcholine
PE: Phosphatidylethanolamine
PEDS1: Plasmanylethanolamine desaturase
PG: Prostaglandin
PTGS: Prostaglandin G/H synthase
PUFA: Polyunsaturated fatty acids
S.tm: *Salmonella enterica* serovar Typhimurium
SACM: *Staphylococcus aureus*-conditioned medium
sWAT: Subcutaneous white adipose tissue
TX: Thromboxane
UPLC-MS/MS: Ultra-performance liquid chromatography‒tandem mass spectrometry
vWAT: Visceral white adipose tissue
WT: Wildtype

## References

[1] Dean JM, Lodhi IJ. Structural and functional roles of ether lipids. Protein Cell. 2018;9:196–206. 28523433 10.1007/s13238-017-0423-5 PMC5818364

[2] Honsho M, Asaoku S, Fujiki Y. Posttranslational regulation of fatty acyl-CoA reductase 1, Far1, controls ether glycerophospholipid synthesis. J Biol Chem. 2010;285:8537–42. 20071337 10.1074/jbc.M109.083311 PMC2838275

[3] Watschinger K, Keller MA, Golderer G, Hermann M, Maglione M, Sarg B, et al. Identification of the gene encoding alkylglycerol monooxygenase defines a third class of tetrahydrobiopterin-dependent enzymes. Proc Natl Acad Sci U S A. 2010;107:13672–7. 20643956 10.1073/pnas.1002404107 PMC2922233

[4] Werner ER, Keller MA, Sailer S, Lackner K, Koch J, Hermann M, et al. The *TMEM189* gene encodes plasmanylethanolamine desaturase which introduces the characteristic vinyl ether double bond into plasmalogens. Proc Natl Acad Sci U S A. 2020;117:7792–8. 32209662 10.1073/pnas.1917461117 PMC7149458

[5] Rizzo WB, Heinz E, Simon M, Craft DA. Microsomal fatty aldehyde dehydrogenase catalyzes the oxidation of aliphatic aldehyde derived from ether glycerolipid catabolism: implications for Sjögren-Larsson syndrome. Biochim Biophys Acta. 2000;1535:1–9. 11113626 10.1016/s0925-4439(00)00077-6

[6] Tietz A, Lindberg M, Kennedy EP. A new pteridine-requiring enzyme system for the oxidation of glyceryl ethers. J Biol Chem. 1964;239:4081–90. 14247652

[7] Watschinger K, Keller MA, McNeill E, Alam MT, Lai S, Sailer S, et al. Tetrahydrobiopterin and alkylglycerol monooxygenase substantially alter the murine macrophage lipidome. Proc Natl Acad Sci U S A. 2015;112:2431. 25675482 10.1073/pnas.1414887112 PMC4345615

[8] Marquet S, Bucheton B, Reymond C, Argiro L, El-Safi SH, Kheir MM, et al. Exome sequencing identifies two variants of the alkylglycerol monooxygenase gene as a cause of relapses in visceral leishmaniasis in children, in Sudan. J Infect Dis. 2017;216:22–8. 28586473 10.1093/infdis/jix277

[9] Alrayes N, Mohamoud HSA, Ahmed S, Almramhi MM, Shuaib TM, Wang J, et al. The alkylglycerol monooxygenase (AGMO) gene previously involved in autism also causes a novel syndromic form of primary microcephaly in a consanguineous Saudi family. J Neurol Sci. 2016;363:240–4. 27000257 10.1016/j.jns.2016.02.063

[10] Okur V, Watschinger K, Niyazov D, McCarrier J, Basel D, Hermann M, et al. Biallelic variants in AGMO with diminished enzyme activity are associated with a neurodevelopmental disorder. Hum Genet. 2019;138:1259–66. 31555905 10.1007/s00439-019-02065-x

[11] Sebat J, Lakshmi B, Malhotra D, Troge J, Lese-Martin C, Walsh T, et al. Strong association of de novo copy number mutations with autism. Science. 2007;316:445–9. 17363630 10.1126/science.1138659 PMC2993504

[12] Fakhro KA, Choi M, Ware SM, Belmont JW, Towbin JA, Lifton RP, et al. Rare copy number variations in congenital heart disease patients identify unique genes in left-right patterning. Proc Natl Acad Sci U S A. 2011;108:2915–20. 21282601 10.1073/pnas.1019645108 PMC3041108

[13] Sailer S, Keller MA, Werner ER, Watschinger K. The emerging physiological role of AGMO 10 years after its gene identification. Life (Basel). 2021;11:88. 33530536 10.3390/life11020088 PMC7911779

[14] Sailer S, Coassin S, Lackner K, Fischer C, McNeill E, Streiter G, et al. When the genome bluffs: a tandem duplication event during generation of a novel agmo knockout mouse model fools routine genotyping. Cell Biosci. 2021;11:1–10. 33726865 10.1186/s13578-021-00566-9 PMC7962373

[15] Papin M, Bouchet AM, Chantôme A, Vandier C. Ether-lipids and cellular signaling: a differential role of alkyl- and alkenyl-ether-lipids? Biochimie. 2023;215:50–9. 37678745 10.1016/j.biochi.2023.09.004

[16] Bazan HE, Tao Y, DeCoster MA, Bazan NG. Platelet-activating factor induces cyclooxygenase-2 gene expression in corneal epithelium. Requirement of calcium in the signal transduction pathway. Invest Ophthalmol Vis Sci. 1997;38:2492–501. 9375567

[17] Salamonsen LA. Effect of platelet activating factor on prostaglandin release from ovine endometrial cells in culture. Prostaglandins Leukot Essent Fat Acids. 1991;44:67–70. 10.1016/0952-3278(91)90147-w 1946564

[18] Na H-K, Inoue H, Surh Y-J. ET-18-O-CH3-induced apoptosis is causally linked to COX-2 upregulation in H-ras transformed human breast epithelial cells. FEBS Lett. 2005;579:6279–87. 16253239 10.1016/j.febslet.2005.09.094

[19] Magnusson CD, Haraldsson GG. Ether lipids. Chem Phys Lipids. 2011;164:315–40. 21635876 10.1016/j.chemphyslip.2011.04.010

[20] Dorninger F, Forss-Petter S, Wimmer I, Berger J. Plasmalogens, platelet-activating factor and beyond - ether lipids in signaling and neurodegeneration. Neurobiol Dis. 2020;145:105061. 32861763 10.1016/j.nbd.2020.105061 PMC7116601

[21] Nagan N, Zoeller RA. Plasmalogens: biosynthesis and functions. Prog Lipid Res. 2001;40:199–229. 11275267 10.1016/s0163-7827(01)00003-0

[22] Dorninger F, Werner ER, Berger J, Watschinger K. Regulation of plasmalogen metabolism and traffic in mammals: the fog begins to lift. Front Cell Dev Biol. 2022;10:946393. 36120579 10.3389/fcell.2022.946393 PMC9471318

[23] Valentine WJ, Yanagida K, Kawana H, Kono N, Noda NN, Aoki J, et al. Update and nomenclature proposal for mammalian lysophospholipid acyltransferases, which create membrane phospholipid diversity. J Biol Chem. 2022;298:101470. 34890643 10.1016/j.jbc.2021.101470 PMC8753187

[24] Tokuoka SM, Kita Y, Shindou H, Shimizu T. Alkylglycerol monooxygenase as a potential modulator for PAF synthesis in macrophages. Biochem Biophys Res Commun. 2013;436:306–12. 23743196 10.1016/j.bbrc.2013.05.099

[25] Yamamoto N, Ngwenya BZ. Activation of mouse peritoneal macrophages by lysophospholipids and ether derivatives of neutral lipids and phospholipids. Cancer Res. 1987;47:2008–13. 2950993

[26] Rangholia N, Leisner TM, Holly SP. Bioactive ether lipids: primordial modulators of cellular signaling. Metabolites. 2021;11:41. 33430006 10.3390/metabo11010041 PMC7827237

[27] Hayashi D, Mouchlis VD, Dennis EA. Each phospholipase A type exhibits distinct selectivity toward sn-1 ester, alkyl ether, and vinyl ether phospholipids. Biochim Biophys Acta Mol Cell Biol Lipids. 2022;1867:159067. 34634490 10.1016/j.bbalip.2021.159067 PMC9188868

[28] Bennett M, Gilroy DW. Lipid mediators in inflammation. Microbiol Spectr. 2016;4. 10.1128/microbiolspec.MCHD-0035-2016 27837747

[29] Dyall SC, Balas L, Bazan NG, Brenna JT, Chiang N, da Costa Souza F, et al. Polyunsaturated fatty acids and fatty acid-derived lipid mediators: recent advances in the understanding of their biosynthesis, structures, and functions. Prog Lipid Res. 2022;86:101165. 35508275 10.1016/j.plipres.2022.101165 PMC9346631

[30] Shimizu T. Lipid mediators in health and disease: enzymes and receptors as therapeutic targets for the regulation of immunity and inflammation. Annu Rev Pharmacol Toxicol. 2009;49:123–50. 18834304 10.1146/annurev.pharmtox.011008.145616

[31] Simmons DL, Botting RM, Hla T. Cyclooxygenase isozymes: the biology of prostaglandin synthesis and inhibition. Pharmacol Rev. 2004;56:387–437. 15317910 10.1124/pr.56.3.3

[32] Rouzer CA, Marnett LJ. Cyclooxygenases: structural and functional insights. J Lipid Res. 2009;50:29–34. Suppl:S. 10.1194/jlr.R800042-JLR200 PMC2674713 18952571

[33] Schoenthaler M, Waltl L, Hasenoehrl T, Seher D, Lutz A, Aulinger L, et al. Novel thiazolopyridine derivatives of diflapolin as dual sEH/FLAP inhibitors with improved solubility. Bioorg Chem. 2023;139:106685. 37418786 10.1016/j.bioorg.2023.106685

[34] Dalli J, Serhan CN. Specific lipid mediator signatures of human phagocytes: microparticles stimulate macrophage efferocytosis and pro-resolving mediators. Blood. 2012;120:e60–72. 22904297 10.1182/blood-2012-04-423525 PMC3471524

[35] Rao Z, Brunner E, Giszas B, Iyer-Bierhoff A, Gerstmeier J, Börner F, et al. Glucocorticoids regulate lipid mediator networks by reciprocal modulation of 15-lipoxygenase isoforms affecting inflammation resolution. Proc Natl Acad Sci U S A. 2023;120:e2302070120. 37603745 10.1073/pnas.2302070120 PMC10469032

[36] Werner ER, Hermetter A, Prast H, Golderer G, Werner-Felmayer G. Widespread occurrence of glyceryl ether monooxygenase activity in rat tissues detected by a novel assay. J Lipid Res. 2007;48:1422–7. 17303893 10.1194/jlr.D600042-JLR200 PMC2851153

[37] Keller MA, Watschinger K, Golderer G, Maglione M, Sarg B, Lindner HH, et al. Monitoring of fatty aldehyde dehydrogenase by formation of pyrenedecanoic acid from pyrenedecanal. J Lipid Res. 2010;51:1554–9. 19965611 10.1194/jlr.D002220 PMC3035519

[38] Jordan PM, Gerstmeier J, Pace S, Bilancia R, Rao Z, Börner F, et al. Staphylococcus aureus-derived α-hemolysin evokes generation of specialized pro-resolving mediators promoting inflammation resolution. Cell Rep. 2020;33:108247. 33053344 10.1016/j.celrep.2020.108247 PMC7729929

[39] Neukirch K, Alsabil K, Dinh C-P, Bilancia R, Raasch M, Ville A, et al. Exploration of long-chain vitamin E metabolites for the discovery of a highly potent, orally effective, and metabolically stable 5-LOX inhibitor that limits inflammation. J Med Chem. 2021;64:11496–526. 34279935 10.1021/acs.jmedchem.1c00806 PMC8365602

[40] Waltl L, Speck K, Wildermuth R, Haut F-L, Permann S, D’Avino D et al. Reorganization of innate immune cell lipid profiles by bioinspired meroterpenoids to limit inflammation. bioRxiv. 2024:2024.05.24.595516. 10.1002/advs.76861 PMC13483160 42610641

[41] Salamone S, Waltl L, Pompignan A, Grassi G, Chianese G, Koeberle A, et al. Phytochemical characterization of L. Chemotype V reveals three new dihydrophenanthrenoids that favorably reprogram lipid mediator biosynthesis in macrophages. Plants (Basel). 2022;11:2130. 36015434 10.3390/plants11162130 PMC9414986

[42] Bligh EG, Dyer WJ. A rapid method of total lipid extraction and purification. Can J Biochem Physiol. 1959;37:911–7. 13671378 10.1139/o59-099

[43] Pein H, Ville A, Pace S, Temml V, Garscha U, Raasch M, et al. Endogenous metabolites of vitamin E limit inflammation by targeting 5-lipoxygenase. Nat Commun. 2018;9:3834. 30237488 10.1038/s41467-018-06158-5 PMC6148290

[44] Pfeifhofer-Obermair C, Brigo N, Tymoszuk P, Weiss AG. A mouse infection model with a wildtype salmonella enterica serovar typhimurium strain for the analysis of inflammatory innate immune cells. Bio Protoc. 2022;12:e4378. 35530516 10.21769/BioProtoc.4378 PMC9018427

[45] Sorgi CA, Zarini S, Martin SA, Sanchez RL, Scandiuzzi RF, Gijón MA, et al. Dormant 5-lipoxygenase in inflammatory macrophages is triggered by exogenous arachidonic acid. Sci Rep. 2017;7:10981. 28887514 10.1038/s41598-017-11496-3 PMC5591212

[46] Werz O, Gerstmeier J, Libreros S, De la Rosa X, Werner M, Norris PC, et al. Human macrophages differentially produce specific resolvin or leukotriene signals that depend on bacterial pathogenicity. Nat Commun. 2018;9:59. 29302056 10.1038/s41467-017-02538-5 PMC5754355

[47] Yao C, Narumiya S. Prostaglandin-cytokine crosstalk in chronic inflammation. Br J Pharmacol. 2019;176:337–54. 30381825 10.1111/bph.14530 PMC6329627

[48] Shapouri-Moghaddam A, Mohammadian S, Vazini H, Taghadosi M, Esmaeili S-A, Mardani F, et al. Macrophage plasticity, polarization, and function in health and disease. J Cell Physiol. 2018;233:6425–40. 29319160 10.1002/jcp.26429 PMC13482560

[49] Ricciotti E, FitzGerald GA. Prostaglandins and inflammation. Arterioscler Thromb Vasc Biol. 2011;31:986–1000. 21508345 10.1161/ATVBAHA.110.207449 PMC3081099

[50] Koeberle A, Werz O. Natural products as inhibitors of prostaglandin E and pro-inflammatory 5-lipoxygenase-derived lipid mediator biosynthesis. Biotechnol Adv. 2018;36:1709–23. 29454981 10.1016/j.biotechadv.2018.02.010

[51] Turini ME, DuBois RN. Cyclooxygenase-2: a therapeutic target. Annu Rev Med. 2002;53:35–57. 11818462 10.1146/annurev.med.53.082901.103952

[52] Mohammed NA, Abd El-Aleem SA, El-Hafiz HA, McMahon RFT. Distribution of constitutive (COX-1) and inducible (COX-2) cyclooxygenase in postviral human liver cirrhosis: a possible role for COX-2 in the pathogenesis of liver cirrhosis. J Clin Pathol. 2004;57:350–4. 15047734 10.1136/jcp.2003.012120 PMC1770276

[53] Chan P-C, Hsiao F-C, Chang H-M, Wabitsch M, Hsieh PS. Importance of adipocyte cyclooxygenase-2 and prostaglandin E2-prostaglandin E receptor 3 signaling in the development of obesity-induced adipose tissue inflammation and insulin resistance. FASEB J. 2016;30:2282–97. 26932930 10.1096/fj.201500127

[54] Motiño O, Agra N, Brea Contreras R, Domínguez-Moreno M, García-Monzón C, Vargas-Castrillón J, et al. Cyclooxygenase-2 expression in hepatocytes attenuates non-alcoholic steatohepatitis and liver fibrosis in mice. Biochim Biophys Acta. 2016;1862:1710–23. 27321932 10.1016/j.bbadis.2016.06.009

[55] Halter F, Tarnawski AS, Schmassmann A, Peskar BM. Cyclooxygenase 2-implications on maintenance of gastric mucosal integrity and ulcer healing: controversial issues and perspectives. Gut. 2001;49:443–53. 11511570 10.1136/gut.49.3.443 PMC1728453

[56] Harris RC. COX-2 and the kidney. J Cardiovasc Pharmacol. 2006;47(Suppl 1):S37–42. 16785827 10.1097/00005344-200605001-00007

[57] Fontaine D, Figiel S, Félix R, Kouba S, Fromont G, Mahéo K, et al. Roles of endogenous ether lipids and associated PUFAs in the regulation of ion channels and their relevance for disease. J Lipid Res. 2020;61:840–58. 32265321 10.1194/jlr.RA120000634 PMC7269763

[58] Chilton FH, Fonteh AN, Surette ME, Triggiani M, Winkler JD. Control of arachidonate levels within inflammatory cells. Biochim Biophys Acta. 1996;1299:1–15. 8555241 10.1016/0005-2760(95)00169-7

[59] Rao Z, Pace S, Jordan PM, Bilancia R, Troisi F, Börner F, et al. Vacuolar (H)-ATPase critically regulates specialized proresolving mediator pathways in human M2-like monocyte-derived macrophages and has a crucial role in resolution of inflammation. J Immunol. 2019;203:1031–43. 31300512 10.4049/jimmunol.1900236 PMC7347291

[60] Shay AE, Diwakar BT, Guan B-J, Narayan V, Urban JF Jr, Prabhu KS. IL-4 up-regulates cyclooxygenase-1 expression in macrophages. J Biol Chem. 2017;292:14544–55. 28684424 10.1074/jbc.M117.785014 PMC5582846

[61] Martinez FO, Gordon S, Locati M, Mantovani A. Transcriptional profiling of the human monocyte-to-macrophage differentiation and polarization: new molecules and patterns of gene expression. J Immunol. 2006;177:7303–11. 17082649 10.4049/jimmunol.177.10.7303

[62] Waqas SFH, Hoang AC, Lin Y-T, Ampem G, Azegrouz H, Balogh L, et al. Neuropeptide FF increases M2 activation and self-renewal of adipose tissue macrophages. J Clin Invest. 2017;127:2842–54. 28581443 10.1172/JCI90152 PMC5490745

[63] Bozelli JC Jr, Azher S, Epand RM. Plasmalogens and chronic inflammatory diseases. Front Physiol. 2021;12:730829. 34744771 10.3389/fphys.2021.730829 PMC8566352

[64] Snyder F. The ether lipid trail: a historical perspective. Biochim Biophys Acta. 1999;1436:265–78. 9989259 10.1016/s0005-2760(98)00172-6

[65] Wey HE. Phorbol diester enhances calcium ionophore A23187-induced [3H]acetate incorporation into platelet-activating factor in murine macrophages: predominant incorporation into 1-O-acyl-2-acetyl-sn-glycero-3-phosphocholine. J Cell Biochem. 1989;39:305–13. 2496135 10.1002/jcb.240390310

[66] Elstad MR, Stafforini DM, McIntyre TM, Prescott SM, Zimmerman GA. Platelet-activating factor acetylhydrolase increases during macrophage differentiation. A novel mechanism that regulates accumulation of platelet-activating factor. J Biol Chem. 1989;264:8467–70. 2722780

[67] Paul S, Smith AAT, Culham K, Gunawan KA, Weir JM, Cinel MA, et al. Shark liver oil supplementation enriches endogenous plasmalogens and reduces markers of dyslipidemia and inflammation. J Lipid Res. 2021;62:100092. 34146594 10.1016/j.jlr.2021.100092 PMC8281607

[68] Qian L, Zhang M, Wu S, Zhong Y, Van Tol E, Cai W. Alkylglycerols modulate the proliferation and differentiation of non-specific agonist and specific antigen-stimulated splenic lymphocytes. PLoS ONE. 2014;9:e96207. 24763671 10.1371/journal.pone.0096207 PMC3999215

[69] Zou Y, Henry WS, Ricq EL, Graham ET, Phadnis VV, Maretich P, et al. Plasticity of ether lipids promotes ferroptosis susceptibility and evasion. Nature. 2020;585:603–8. 32939090 10.1038/s41586-020-2732-8 PMC8051864

[70] Yang WS, SriRamaratnam R, Welsch ME, Shimada K, Skouta R, Viswanathan VS, et al. Regulation of ferroptotic cancer cell death by GPX4. Cell. 2014;156:317–31. 24439385 10.1016/j.cell.2013.12.010 PMC4076414

[71] Pontsler AV, St Hilaire A, Marathe GK, Zimmerman GA, McIntyre TM. Cyclooxygenase-2 is induced in monocytes by peroxisome proliferator activated receptor gamma and oxidized alkyl phospholipids from oxidized low density lipoprotein. J Biol Chem. 2002;277:13029–36. 11809750 10.1074/jbc.M109546200

[72] Zhu Y, Liu X-J, Yang P, Zhao M, Lv L-X, Zhang G-D, et al. Alkylglyceronephosphate synthase (AGPS) alters lipid signaling pathways and supports chemotherapy resistance of glioma and hepatic carcinoma cell lines. Asian Pac J Cancer Prev. 2014;15:3219–26. 24815474 10.7314/apjcp.2014.15.7.3219

[73] Lipoldová M, Demant P. Gene-specific sex effects on susceptibility to infectious diseases. Front Immunol. 2021;12:712688. 34721380 10.3389/fimmu.2021.712688 PMC8553003

[74] Klein SL, Flanagan KL. Sex differences in immune responses. Nat Rev Immunol. 2016;16:626–38. 27546235 10.1038/nri.2016.90

[75] Banhos Danneskiold-Samsøe N, Sonne SB, Larsen JM, Hansen AN, Fjære E, Isidor MS, et al. Overexpression of cyclooxygenase-2 in adipocytes reduces fat accumulation in inguinal white adipose tissue and hepatic steatosis in high-fat fed mice. Sci Rep. 2019;9:8979. 31222118 10.1038/s41598-019-45062-w PMC6586826

[76] Pan Y, Cao S, Tang J, Arroyo JP, Terker AS, Wang Y, et al. Cyclooxygenase-2 in adipose tissue macrophages limits adipose tissue dysfunction in obese mice. J Clin Invest. 2022;132:e152391. 35499079 10.1172/JCI152391 PMC9057601

[77] Ghoshal S, Trivedi DB, Graf GA, Loftin CD. Cyclooxygenase-2 deficiency attenuates adipose tissue differentiation and inflammation in mice. J Biol Chem. 2011;286:889–98. 20961858 10.1074/jbc.M110.139139 PMC3013048

[78] Pace S, Rossi A, Krauth V, Dehm F, Troisi F, Bilancia R, et al. Sex differences in prostaglandin biosynthesis in neutrophils during acute inflammation. Sci Rep. 2017;7:3759. 28630405 10.1038/s41598-017-03696-8 PMC5476623

[79] Shen BQ, Sankaranarayanan I, Price TJ, Tavares-Ferreira D. Sex-differences in prostaglandin signaling: a semi-systematic review and characterization of PTGDS expression in human sensory neurons. Sci Rep. 2023;13:4670. 36949072 10.1038/s41598-023-31603-x PMC10033690

