# Supplementary material for: Alkylglycerol monooxygenase represses prostanoid biosynthesis in a sex-dependent manner
Source: Cell Biosci. 2025 Jun 5;15:80. doi: 10.1186/s13578-025-01419-5 (PMC12143016; doi:10.1186/s13578-025-01419-5)
Supplement: Supplementary file 1 — Supplementary Material 1 [file 13578_2025_1419_MOESM1_ESM.docx]

# Supplementary Figures


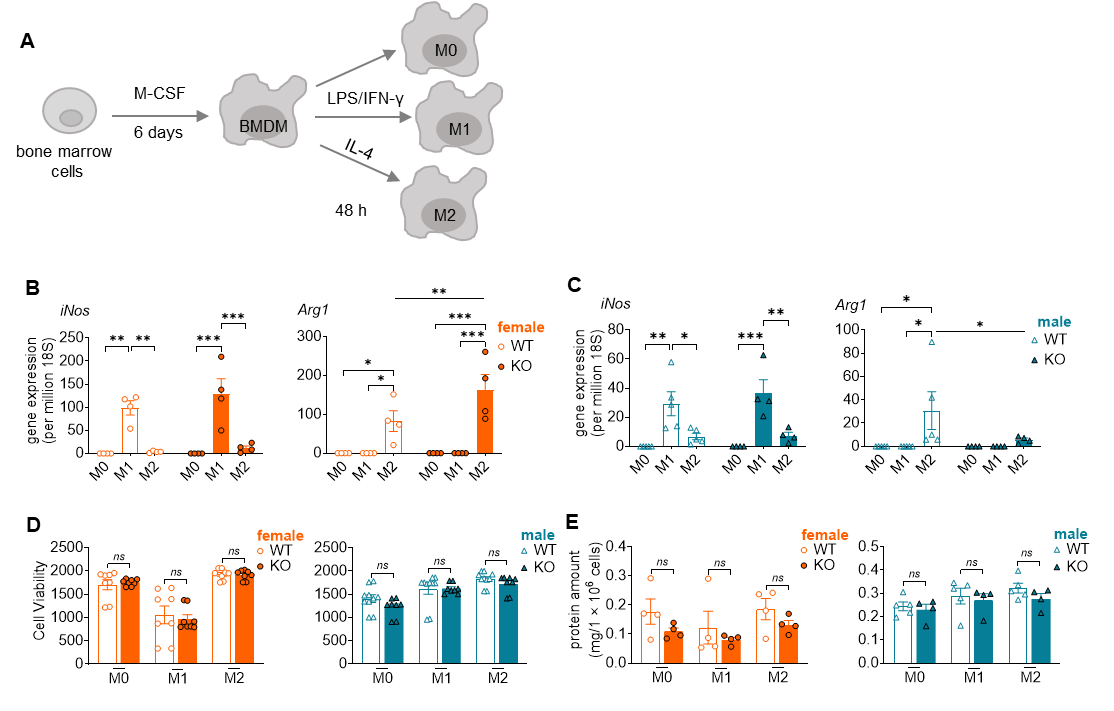
Fig. S1 Polarization of BMDMs to the M1 and M2 phenotypes

(A) Schematic overview about the polarization of murine BMDMs towards the M0, M1 and M2 phenotypes. B-E) Murine BMDMs isolated from wildtype (WT, open circles/triangles) or *Agmo* knockout (KO, filled circles/triangles) mice (female, orange; male, blue) were polarized to the M0, M1 or M2 phenotypes. B-C) The expression of *iNos* and *Arg1* in M0, M1 or M2 from B) female and C) male mice. (D) Cell viability of the polarized M0, M1 and M2 measured by cell titer blue reagent. (E) Protein amount of the polarized M0, M1 and M2 measured by DC protein assay. Data are shown as mean ± s.e.m; *n* = 4–5. ****p* < 0.001, ***p* < 0.01, **p* < 0.05, ns: not significant; B-C) ordinary two-way ANOVA plus Tukey *post hoc* tests or D-E) two-tailed unpaired *t*-test.


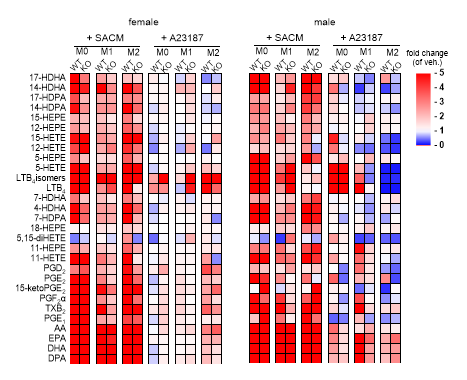


Fig. S2 SACM stimulates lipid mediator production in BMDMs

Murine BMDMs isolated from wildtype (WT) or *Agmo* knockout (KO) mice were polarized to the M0, M1 or M2 phenotypes, and then stimulated with vehicle (veh., DMSO, 0.1%), *S. aureus*-conditioned medium (SACM, 1% v/v, 3 h) or Ca^2+^-ionophore A23187 (2.5 µM, 15 min). Lipid mediators were extracted and analyzed by UPLC-MS/MS. Color codes indicate the fold change against vehicle control (veh.). Data are shown as mean; *n* = 4–5.


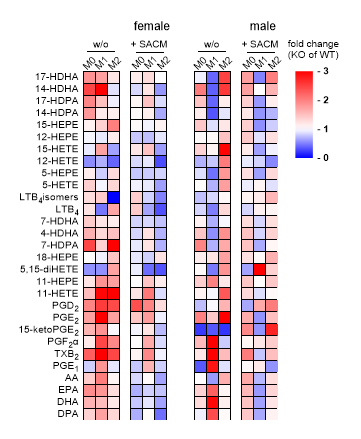


Fig. S3 Effect of *Agmo* knockout on lipid mediator profile in BMDMs

Murine BMDMs isolated from wildtype (WT) or *Agmo* knockout (KO) mice were polarized to the M0, M1 or M2 phenotypes, and then stimulated with vehicle (veh., DMSO, 0.1%), *S. aureus*-conditioned medium (SACM, 1% v/v, 3 h). Lipid mediators were extracted and analyzed by UPLC-MS/MS. Color codes indicate the fold change of KO against WT. Data are shown as mean; *n* = 4–5.


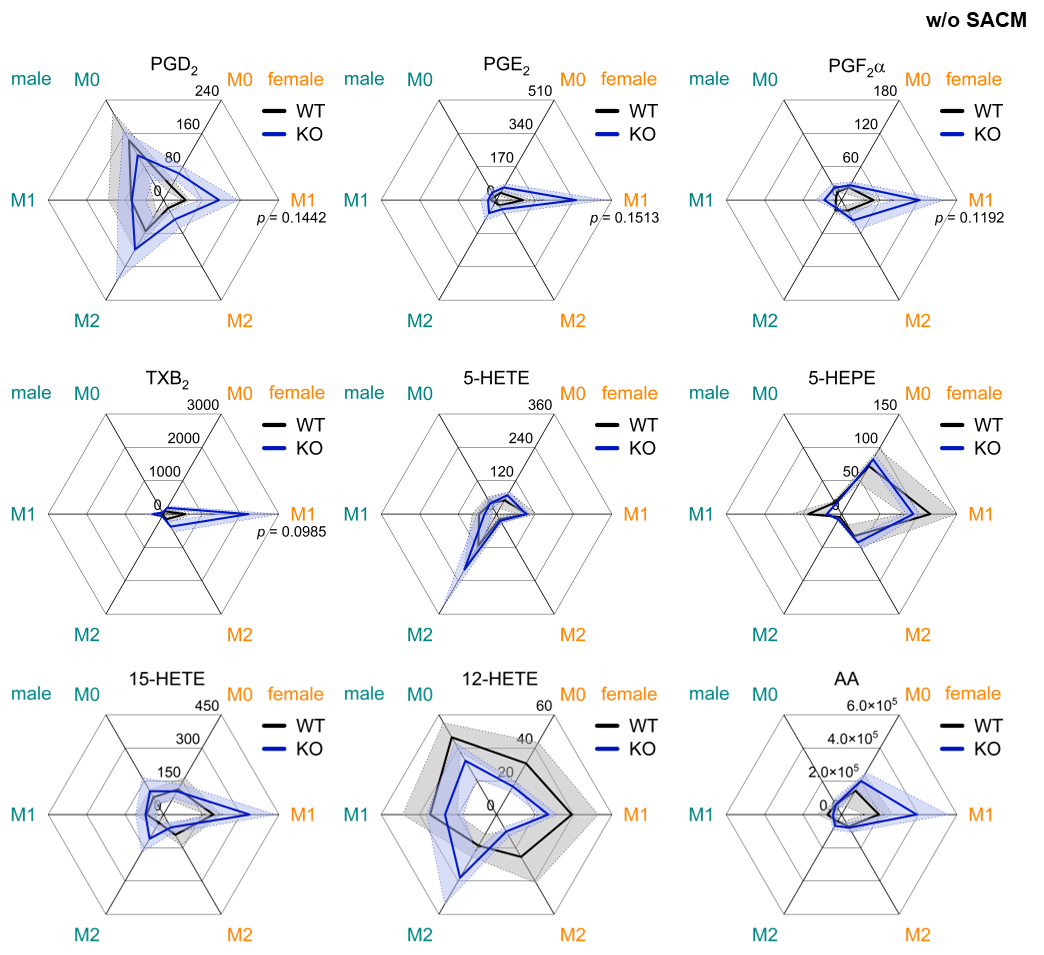
Fig. S4 *Agmo* knockout elevates the formation of COX metabolites in non-stimulated M1 BMDMs.

Murine BMDMs isolated from wildtype (WT) or *Agmo* knockout (KO) mice (female, orange; male, blue) were polarized to the M0, M1 or M2 phenotypes, and then incubated in PBS + Ca^2+^ (1 mM) for 3 h. Lipid mediators were extracted and analyzed by UPLC-MS/MS. A) The levels of exemplary lipid mediators (pg/0.2 mg protein) are shown in radar-plots. Data are shown as mean ± s.e.m, the mean values were connected by solid lines (WT, black; KO, blue) and the s.e.m values were connected by dashed lines (WT, black; KO, blue). *n* = 4–5. **p* < 0.05; two tailed unpaired *t*-tests.


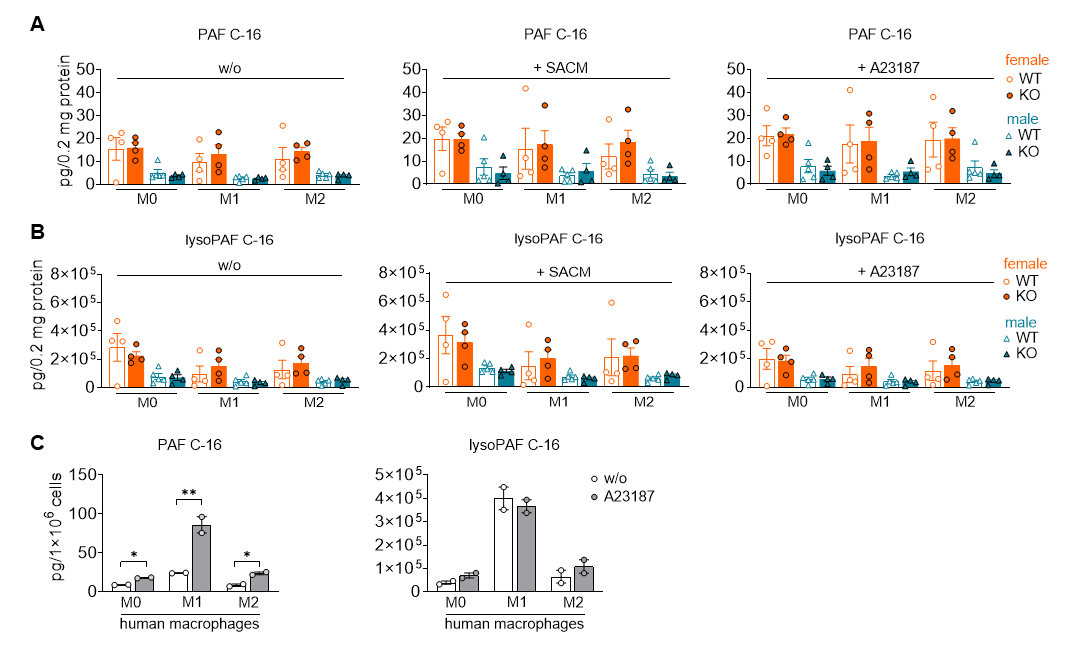
Fig. S5 *Agmo* knockout does not alter PAF and lyso-PAF levels in BMDMs

Murine BMDMs isolated from wildtype (WT, open circles/triangles) or *Agmo* knockout (KO, filled circles/triangles) mice (female, orange; male, blue) or human macrophages differentiated from PBMC were polarized to the M0, M1 or M2 phenotypes, and then stimulated with vehicle (DMSO, 0.1% v/v), *S. aureus*-conditioned medium (SACM, 1% v/v, 3 h) or Ca^2+^-ionophore A23187 (2.5 µM, 15 min). Lipids were extracted, PAF C-16 and lyso-PAF C-16 were analyzed by UPLC-MS/MS. The levels of PAF C-16 and lyso-PAF C-16 in A, B) BMDMs or C) human macrophages were shown. Data are shown as mean ± s.e.m; A, B) *n* = 4–5 and C) *n* = 2.


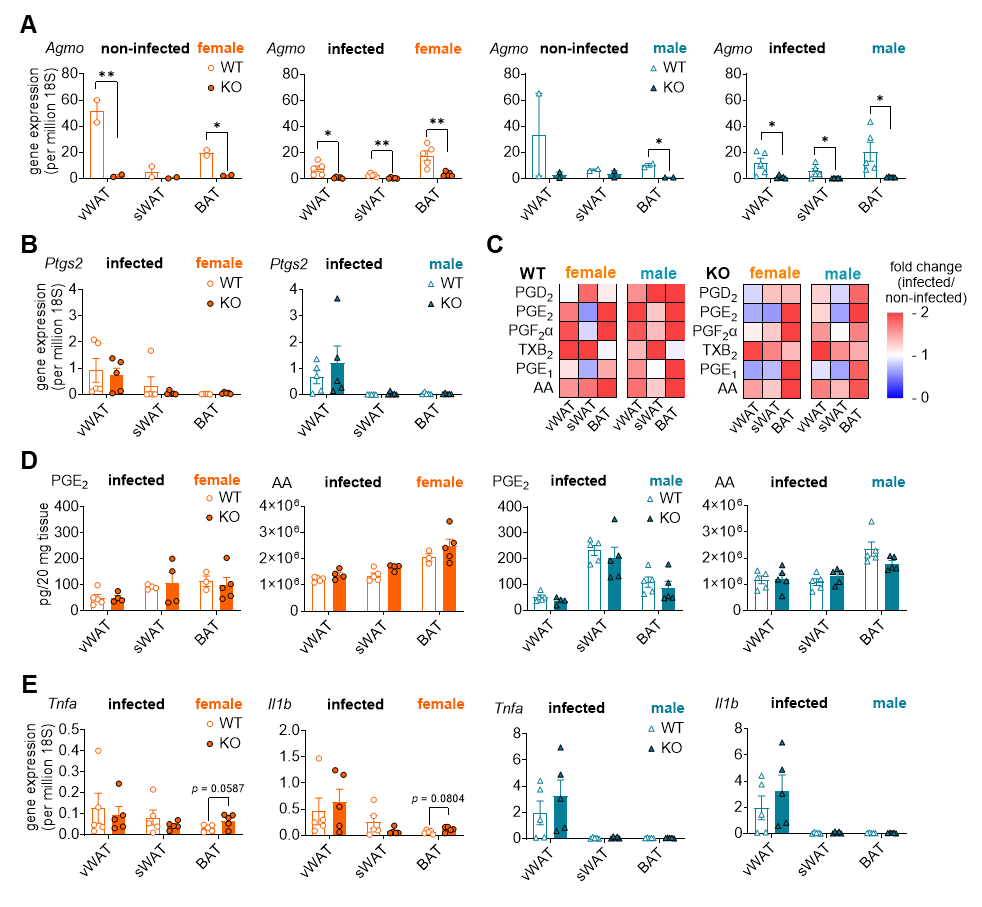
Fig. S6 Effect of AGMO on *Ptgs2*, cytokine expression and COX product formation in adipose tissues.

Female and male wildtype (WT, open circles/triangles) or *Agmo* knockout (KO, open circles/triangles) mice (female, orange; male, blue) were not treated (non-infected) or challenged with *Salmonella* *enterica* serovar Typhimurium (S.tm) ATCC14028 wildtype strain (infected) for 3 days, before adipose tissues (vWAT, sWAT and BAT) were collected and homogenized. A-B, E) mRNA levels of A) *Agmo*, B) *Ptgs2*, E) *Tnfa,* and *Il1b*. C-D) Lipid mediators were extracted and analyzed by UPLC-MS/MS. C) Heat-map shows the changes of COX metabolites and arachidonic acid (AA) in the mice tissues. Color codes indicate the fold change of the lipid mediators in infected tissue against non-infected tissue. D) Exemplary levels of PGE_2_ and AA. Data are shown as A-B, D-E) mean ± s.e.m; or C) mean; *n* = 2 (panel A, uninfected) or *n* = 5; **p* < 0.05, ***p* < 0.01; two-tailed unpaired *t-*tests.

Supplementary Tables

**Table. S1 Lipid mediator profile in non-stimulated BMDMs from female mice.**

|  | M0_WT | M0_KO | M1_WT | M1_KO | M2_WT | M2_KO |
| --- | --- | --- | --- | --- | --- | --- |
| 17-HDHA | 28 ± 19 | 53 ± 13 | 62 ± 25 | 109 ± 20 | 23 ± 20 | 27 ± 12 |
| 14-HDHA | 4 ± 4 | 11 ± 3 | 5 ± 3 | 16 ± 5 | 3 ± 2 | 3 ± 1 |
| 17-HDPA | 32 ± 18 | 63 ± 10 | 62 ± 20 | 92 ± 18 | 19 ± 14 | 18 ± 6 |
| 14-HDPA | 22 ± 16 | 40 ± 10 | 32 ± 9 | 36 ± 6 | 7 ± 5 | 8 ± 3 |
| 15-HEPE | 24 ± 8 | 26 ± 2 | 33 ± 8 | 45 ± 3 | 11 ± 7 | 22 ± 5 |
| 12-HEPE | 14 ± 6 | 14 ± 3 | 17 ± 4 | 16 ± 0 | 6 ± 2 | 7 ± 2 |
| 15-HETE | 114 ± 52 | 105 ± 31 | 195 ± 29 | 336 ± 102 | 92 ± 47 | 58 ± 23 |
| 12-HETE | 31 ± 13 | 17 ± 3 | 39 ± 14 | 27 ± 5 | 25 ± 16 | 10 ± 3 |
| 5-HEPE | 72 ± 26 | 82 ± 6 | 115 ± 35 | 93 ± 9 | 33 ± 17 | 42 ± 10 |
| 5-HETE | 50 ± 30 | 68 ± 13 | 97 ± 28 | 92 ± 11 | 18 ± 12 | 24 ± 9 |
| LTB_4_ isomers | 1 ± 1 | 1 ± 0 | 0 ± 0 | 0 ± 0 | 0 ± 0 | 1 ± 1 |
| LTB_4_ | 1 ± 1 | 2 ± 1 | 2 ± 2 | 1 ± 1 | 1 ± 1 | 1 ± 1 |
| 7-HDHA | 9 ± 3 | 12 ± 3 | 11 ± 2 | 14 ± 1 | 5 ± 2 | 6 ± 1 |
| 4-HDHA | 9 ± 6 | 15 ± 3 | 14 ± 5 | 18 ± 3 | 4 ± 3 | 6 ± 3 |
| 7-HDPA | 24 ± 17 | 59 ± 14 | 59 ± 19 | 81 ± 18 | 11 ± 6 | 33 ± 20 |
| 18-HEPE | 147 ± 42 | 150 ± 17 | 128 ± 41 | 121 ± 20 | 108 ± 62 | 155 ± 41 |
| 5,15-diHETE | 93 ± 54 | 53 ± 20 | 88 ± 34 | 50 ± 13 | 42 ± 21 | 72 ± 27 |
| 11-HEPE | 20 ± 7 | 24 ± 4 | 45 ± 11 | 83 ± 15 | 13 ± 8 | 22 ± 6 |
| 11-HETE | 38 ± 20 | 58 ± 15 | 195 ± 75 | 578 ± 216 | 26 ± 12 | 234 ± 201 |
| PGD_2_ | 33 ± 13 | 64 ± 16 | 46 ± 7 | 116 ± 41 | 19 ± 9 | 47 ± 20 |
| PGE_2_ | 37 ± 16 | 65 ± 18 | 117 ± 44 | 352 ± 136 | 27 ± 18 | 47 ± 21 |
| 15-keto PGE_2_ | 5 ± 3 | 8 ± 2 | 8 ± 2 | 14 ± 4 | 3 ± 2 | 3 ± 1 |
| PGF_2_α | 23 ± 11 | 27 ± 6 | 50 ± 9 | 122 ± 38 | 19 ± 9 | 36 ± 16 |
| TXB_2_ | 83 ± 57 | 187 ± 53 | 561 ± 182 | 2209 ± 824 | 153 ± 108 | 375 ± 158 |
| PGE_1_ | 3 ± 1 | 2 ± 1 | 10 ± 2 | 19 ± 6 | 3 ± 2 | 3 ± 1 |
| AA | 144263  ± 97341 | 202162  ± 51354 | 194172  ± 71193 | 390578  ± 164303 | 72612  ± 34456 | 78743  ± 26946 |
| EPA | 5227  ± 3793 | 9886  ± 2116 | 7550  ± 3174 | 13759  ± 4428 | 2446  ± 1805 | 3079  ± 1022 |
| DHA | 90690  ± 66531 | 144586  ± 27842 | 152254  ± 61733 | 231207  ± 73338 | 38191 ±  22382 | 44941  ± 13144 |
| DPA | 2659  ± 2092 | 3582  ± 733 | 2826  ± 1014 | 4904  ± 1575 | 921  ± 565 | 1208  ± 408 |

Data are shown as mean ± s.e.m; pg/0.2 mg protein. WT: wildtype; KO: *Agmo*-deficient.

**Table. S2 Lipid mediator profile in SACM-stimulated BMDMs from female mice.**

|  | M0_WT | M0_KO | M1_WT | M1_KO | M2_WT | M2_KO |
| --- | --- | --- | --- | --- | --- | --- |
| 17-HDHA | 144 ± 61 | 165 ± 27 | 154 ± 55 | 195 ± 29 | 45 ± 21 | 45 ± 10 |
| 14-HDHA | 59 ± 25 | 50 ± 9 | 37 ± 13 | 44 ± 5 | 18 ± 10 | 10 ± 3 |
| 17-HDPA | 121 ± 54 | 129 ± 21 | 144 ± 38 | 213 ± 28 | 59 ± 30 | 51 ± 8 |
| 14-HDPA | 106 ± 51 | 109 ± 18 | 86 ± 24 | 95 ± 4 | 39 ± 19 | 26 ± 5 |
| 15-HEPE | 52 ± 10 | 53 ± 10 | 64 ± 14 | 61 ± 11 | 32 ± 10 | 36 ± 6 |
| 12-HEPE | 38 ± 4 | 30 ± 4 | 39 ± 8 | 29 ± 5 | 22 ± 6 | 20 ± 5 |
| 15-HETE | 546 ± 161 | 649 ± 96 | 530 ± 90 | 776 ± 129 | 569 ± 175 | 348 ± 72 |
| 12-HETE | 119 ± 26 | 101 ± 20 | 91 ± 9 | 82 ± 16 | 109 ± 64 | 32 ± 9 |
| 5-HEPE | 206 ± 61 | 160 ± 32 | 183 ± 54 | 145 ± 23 | 116 ± 36 | 95 ± 22 |
| 5-HETE | 527 ± 168 | 497 ± 116 | 233 ± 68 | 212 ± 24 | 205 ± 72 | 127 ± 36 |
| LTB_4_ isomers | 101 ± 29 | 140 ± 58 | 20 ± 7 | 16 ± 5 | 33 ± 10 | 17 ± 6 |
| LTB_4_ | 53 ± 16 | 74 ± 31 | 5 ± 2 | 3 ± 2 | 7 ± 2 | 2 ± 1 |
| 7-HDHA | 28 ± 6 | 23 ± 3 | 23 ± 5 | 19 ± 2 | 15 ± 5 | 11 ± 2 |
| 4-HDHA | 59 ± 24 | 49 ± 11 | 35 ± 10 | 43 ± 4 | 30 ± 14 | 21 ± 6 |
| 7-HDPA | 216 ± 75 | 195 ± 37 | 148 ± 41 | 180 ± 20 | 103 ± 43 | 72 ± 18 |
| 18-HEPE | 170 ± 34 | 154 ± 6 | 188 ± 50 | 154 ± 13 | 203 ± 88 | 246 ± 66 |
| 5,15-diHETE | 46 ± 15 | 42 ± 10 | 99 ± 30 | 41 ± 11 | 112 ± 80 | 37 ± 11 |
| 11-HEPE | 54 ± 12 | 55 ± 5 | 85 ± 20 | 123 ± 19 | 43 ± 15 | 49 ± 10 |
| 11-HETE | 408 ± 119 | 493 ± 78 | 611 ± 166 | 1250 ± 266 | 366 ± 106 | 315 ± 65 |
| PGD_2_ | 98 ± 30 | 233 ± 43 | 101 ± 17 | 198 ± 30 | 93 ± 26 | 118 ± 15 |
| PGE_2_ | 229 ± 82 | 331 ± 62 | 345 ± 87 | 613 ± 112 | 168 ± 72 | 157 ± 30 |
| 15-keto PGE_2_ | 44 ± 14 | 42 ± 10 | 37 ± 12 | 45 ± 9 | 28 ± 11 | 20 ± 4 |
| PGF_2_α | 148 ± 32 | 150 ± 17 | 137 ± 15 | 228 ± 27 | 137 ± 41 | 117 ± 17 |
| TXB_2_ | 2886 ± 666 | 3118 ± 444 | 2817 ± 702 | 5429 ± 724 | 2972 ± 994 | 2819 ± 443 |
| PGE_1_ | 14 ± 3 | 11 ± 2 | 24 ± 3 | 39 ± 9 | 12 ± 3 | 10 ± 3 |
| AA | 4482846  ± 1633156 | 3794239  ± 854430 | 2532541  ± 832084 | 2626906  ± 268957 | 2068430  ± 870212 | 1316487  ± 379385 |
| EPA | 109390  ± 18025 | 74493  ± 15255 | 75680  ± 23570 | 61708  ± 6730 | 44580  ± 17349 | 33755  ± 9278 |
| DHA | 2131242  ± 673512 | 1658762  ± 398198 | 1529169  ± 456039 | 1373562  ± 212934 | 1027891  ± 429289 | 654362  ± 165239 |
| DPA | 78121  ± 31558 | 53172  ± 16993 | 42626  ± 13339 | 43809  ± 5123 | 37572  ± 19079 | 16066  ± 3826 |

Data are shown as mean ± s.e.m; pg/0.2 mg protein. SACM: *S. aureus*-conditioned medium; WT: wildtype; KO: *Agmo*-deficient.

**Table. S3 Lipid mediator profile in Ca^2+^-ionophore A23187-stimulated BMDMs from female mice.**

|  | M0_WT | M0_TG | M1_WT | M1_TG | M2_WT | M2_TG |
| --- | --- | --- | --- | --- | --- | --- |
| 17-HDHA | 38 ± 28 | 52 ± 15 | 52 ± 24 | 185 ± 118 | 12 ± 8 | 21 ± 11 |
| 14-HDHA | 5 ± 4 | 13 ± 4 | 11 ± 5 | 33 ± 26 | 3 ± 2 | 5 ± 2 |
| 17-HDPA | 36 ± 20 | 72 ± 16 | 69 ± 22 | 150 ± 66 | 17 ± 8 | 24 ± 8 |
| 14-HDPA | 19 ± 13 | 52 ± 17 | 33 ± 10 | 58 ± 27 | 6 ± 3 | 5 ± 3 |
| 15-HEPE | 26 ± 11 | 30 ± 3 | 34 ± 8 | 63 ± 20 | 10 ± 6 | 24 ± 9 |
| 12-HEPE | 12 ± 5 | 15 ± 2 | 16 ± 3 | 17 ± 5 | 7 ± 3 | 8 ± 3 |
| 15-HETE | 79 ± 45 | 133 ± 29 | 178 ± 50 | 586 ± 281 | 90 ± 49 | 143 ± 43 |
| 12-HETE | 16 ± 8 | 18 ± 2 | 27 ± 7 | 63 ± 39 | 12 ± 4 | 14 ± 6 |
| 5-HEPE | 72 ± 28 | 87 ± 10 | 114 ± 34 | 102 ± 11 | 40 ± 22 | 43 ± 12 |
| 5-HETE | 41 ± 18 | 75 ± 15 | 120 ± 37 | 175 ± 94 | 38 ± 18 | 40 ± 15 |
| LTB_4_ isomers | 2 ± 1 | 8 ± 3 | 0 ± 0 | 8 ± 7 | 4 ± 2 | 7 ± 4 |
| LTB_4_ | 4 ± 2 | 5 ± 2 | 1 ± 1 | 4 ± 2 | 4 ± 3 | 5 ± 2 |
| 7-HDHA | 9 ± 4 | 12 ± 2 | 14 ± 4 | 12 ± 1 | 6 ± 2 | 7 ± 2 |
| 4-HDHA | 6 ± 3 | 18 ± 5 | 17 ± 5 | 25 ± 11 | 5 ± 3 | 7 ± 2 |
| 7-HDPA | 20 ± 10 | 59 ± 14 | 61 ± 19 | 88 ± 20 | 15 ± 9 | 18 ± 4 |
| 18-HEPE | 146 ± 45 | 150 ± 14 | 133 ± 40 | 146 ± 33 | 128 ± 83 | 175 ± 66 |
| 5,15-diHETE | 40 ± 7 | 52 ± 12 | 76 ± 21 | 110 ± 21 | 57 ± 22 | 62 ± 17 |
| 11-HEPE | 21 ± 10 | 29 ± 3 | 50 ± 12 | 94 ± 23 | 17 ± 12 | 31 ± 9 |
| 11-HETE | 36 ± 19 | 81 ± 16 | 234 ± 100 | 677 ± 276 | 65 ± 45 | 128 ± 39 |
| PGD_2_ | 43 ± 21 | 151 ± 35 | 57 ± 12 | 126 ± 38 | 74 ± 46 | 139 ± 34 |
| PGE_2_ | 27 ± 13 | 76 ± 10 | 179 ± 66 | 394 ± 115 | 56 ± 34 | 83 ± 19 |
| 15-keto PGE_2_ | 5 ± 2 | 12 ± 1 | 12 ± 3 | 19 ± 4 | 8 ± 4 | 11 ± 1 |
| PGF_2_α | 24 ± 9 | 42 ± 7 | 75 ± 17 | 127 ± 30 | 47 ± 23 | 55 ± 11 |
| TXB_2_ | 115 ± 55 | 334 ± 52 | 946 ± 273 | 2710 ± 852 | 533 ± 351 | 1021 ± 264 |
| PGE_1_ | 2 ± 0 | 4 ± 1 | 13 ± 5 | 21 ± 7 | 4 ± 2 | 4 ± 1 |
| AA | 141114  ± 80544 | 323013  ± 78688 | 269253  ± 122090 | 633035  ± 276597 | 216157  ± 136890 | 278303  ± 105484 |
| EPA | 6169  ± 3781 | 11323  ± 2525 | 8478  ± 4225 | 15285  ± 6068 | 5412  ± 3908 | 6735  ± 2653 |
| DHA | 71544  ± 39617 | 163815  ± 35019 | 171335 ± 85066 | 286611  ± 126445 | 74273 ± 45191 | 66684  ± 23418 |
| DPA | 2159  ± 1305 | 4511  ± 1116 | 3769  ± 1705 | 7648  ± 3353 | 1895  ± 1190 | 1736  ± 525 |

Data are shown as mean ± s.e.m; pg/0.2 mg protein. WT: wildtype; KO: *Agmo* knockout.

**Table. S4 Lipid mediator profile in non-stimulated BMDMs from male mice.**

|  | M0_WT | M0_KO | M1_WT | M1_KO | M2_WT | M2_KO |
| --- | --- | --- | --- | --- | --- | --- |
| 17-HDHA | 9 ± 3 | 9 ± 2 | 30 ± 22 | 12 ± 5 | 2 ± 1 | 5 ± 3 |
| 14-HDHA | 2 ± 0 | 4 ± 2 | 5 ± 4 | 2 ± 1 | 0 ± 0 | 1 ± 1 |
| 17-HDPA | 18 ± 4 | 26 ± 8 | 55 ± 40 | 22 ± 6 | 4 ± 2 | 5 ± 1 |
| 14-HDPA | 14 ± 5 | 16 ± 6 | 20 ± 13 | 7 ± 3 | 2 ± 1 | 2 ± 1 |
| 15-HEPE | 9 ± 1 | 13 ± 3 | 12 ± 4 | 10 ± 3 | 2 ± 0 | 3 ± 1 |
| 12-HEPE | 7 ± 1 | 8 ± 0 | 7 ± 1 | 8 ± 1 | 2 ± 0 | 3 ± 1 |
| 15-HETE | 78 ± 32 | 105 ± 61 | 65 ± 43 | 70 ± 38 | 37 ± 25 | 109 ± 62 |
| 12-HETE | 47 ± 9 | 32 ± 11 | 35 ± 14 | 27 ± 10 | 19 ± 7 | 38 ± 16 |
| 5-HEPE | 17 ± 2 | 15 ± 1 | 43 ± 23 | 19 ± 2 | 4 ± 1 | 8 ± 2 |
| 5-HETE | 39 ± 22 | 39 ± 16 | 54 ± 21 | 38 ± 24 | 114 ± 97 | 200 ± 135 |
| LTB_4_ isomers | 6 ± 1 | 4 ± 2 | 6 ± 1 | 5 ± 3 | 23 ± 21 | 24 ± 16 |
| LTB_4_ | 3 ± 1 | 2 ± 1 | 3 ± 1 | 1 ± 0 | 17 ± 15 | 22 ± 19 |
| 7-HDHA | 6 ± 1 | 7 ± 1 | 7 ± 3 | 5 ± 2 | 2 ± 0 | 2 ± 1 |
| 4-HDHA | 3 ± 1 | 5 ± 2 | 7 ± 5 | 4 ± 1 | 1 ± 0 | 2 ± 1 |
| 7-HDPA | 6 ± 1 | 12 ± 6 | 23 ± 18 | 16 ± 8 | 2 ± 1 | 4 ± 1 |
| 18-HEPE | 77 ± 10 | 88 ± 11 | 56 ± 5 | 67 ± 7 | 14 ± 2 | 24 ± 10 |
| 5,15-diHETE | 31 ± 19 | 32 ± 18 | 66 ± 58 | 31 ± 23 | 20 ± 12 | 37 ± 22 |
| 11-HEPE | 8 ± 1 | 11 ± 2 | 9 ± 1 | 17 ± 6 | 3 ± 0 | 4 ± 1 |
| 11-HETE | 19 ± 6 | 20 ± 9 | 41 ± 32 | 39 ± 28 | 8 ± 3 | 17 ± 8 |
| PGD_2_ | 144 ± 67 | 107 ± 60 | 67 ± 47 | 66 ± 29 | 75 ± 45 | 119 ± 76 |
| PGE_2_ | 19 ± 6 | 38 ± 18 | 26 ± 15 | 38 ± 32 | 13 ± 3 | 66 ± 37 |
| 15-keto PGE_2_ | 12 ± 7 | 3 ± 2 | 7 ± 5 | 2 ± 2 | 9 ± 6 | 2 ± 1 |
| PGF_2_α | 14 ± 1 | 22 ± 7 | 9 ± 2 | 27 ± 14 | 19 ± 6 | 15 ± 2 |
| TXB_2_ | 42 ± 18 | 49 ± 26 | 66 ± 31 | 278 ± 206 | 41 ± 13 | 63 ± 29 |
| PGE_1_ | 10 ± 6 | 3 ± 1 | 3 ± 1 | 8 ± 4 | 4 ± 2 | 3 ± 1 |
| AA | 59264  ± 14444 | 59754  ± 27361 | 73530  ± 53326 | 44351  ± 14938 | 41701  ± 7746 | 68004  ± 24069 |
| EPA | 899 ± 182 | 1203 ± 537 | 296 ± 74 | 662 ± 225 | 613 ± 165 | 661 ± 108 |
| DHA | 20062  ± 3734 | 25475  ± 11480 | 8011  ± 2061 | 23540  ± 7810 | 12410  ± 2786 | 13983  ± 1916 |
| DPA | 509 ± 115 | 477 ± 177 | 179 ± 47 | 428 ± 202 | 303 ± 68 | 313 ± 37 |

Data are shown as mean ± s.e.m; pg/0.2 mg protein. WT: wildtype; KO: *Agmo*-deficient.

**Table. S5 Lipid mediator profile in SACM-stimulated BMDMs from male mice.**

|  | M0_WT | M0_KO | M1_WT | M1_KO | M2_WT | M2_KO |
| --- | --- | --- | --- | --- | --- | --- |
| 17-HDHA | 47 ± 8 | 83 ± 8 | 62 ± 31 | 32 ± 9 | 14 ± 4 | 27 ± 13 |
| 14-HDHA | 14 ± 3 | 24 ± 6 | 8 ± 2 | 8 ± 3 | 3 ± 1 | 5 ± 4 |
| 17-HDPA | 44 ± 10 | 61 ± 16 | 76 ± 39 | 45 ± 18 | 17 ± 6 | 19 ± 7 |
| 14-HDPA | 42 ± 9 | 50 ± 13 | 34 ± 13 | 20 ± 4 | 10 ± 5 | 7 ± 4 |
| 15-HEPE | 24 ± 3 | 33 ± 3 | 24 ± 4 | 13 ± 6 | 8 ± 1 | 6 ± 2 |
| 12-HEPE | 16 ± 2 | 23 ± 1 | 18 ± 1 | 16 ± 1 | 5 ± 1 | 7 ± 1 |
| 15-HETE | 217 ± 41 | 252 ± 50 | 204 ± 50 | 128 ± 10 | 108 ± 24 | 101 ± 16 |
| 12-HETE | 72 ± 23 | 66 ± 14 | 53 ± 12 | 44 ± 6 | 48 ± 20 | 26 ± 6 |
| 5-HEPE | 93 ± 14 | 116 ± 14 | 77 ± 17 | 49 ± 7 | 23 ± 6 | 30 ± 6 |
| 5-HETE | 474 ± 189 | 439 ± 186 | 171 ± 61 | 185 ± 78 | 215 ± 110 | 189 ± 115 |
| LTB_4_ isomers | 77 ± 34 | 84 ± 38 | 24 ± 13 | 28 ± 11 | 32 ± 13 | 50 ± 38 |
| LTB_4_ | 28 ± 18 | 23 ± 12 | 7 ± 1 | 7 ± 3 | 20 ± 11 | 22 ± 18 |
| 7-HDHA | 15 ± 2 | 19 ± 1 | 11 ± 3 | 9 ± 1 | 6 ± 1 | 7 ± 2 |
| 4-HDHA | 30 ± 5 | 44 ± 6 | 16 ± 6 | 13 ± 1 | 7 ± 3 | 10 ± 3 |
| 7-HDPA | 76 ± 8 | 111 ± 25 | 63 ± 26 | 42 ± 13 | 18 ± 7 | 19 ± 6 |
| 18-HEPE | 97 ± 12 | 121 ± 14 | 99 ± 11 | 103 ± 12 | 42 ± 10 | 67 ± 10 |
| 5,15-diHETE | 28 ± 11 | 18 ± 8 | 15 ± 6 | 100 ± 89 | 18 ± 6 | 25 ± 15 |
| 11-HEPE | 23 ± 3 | 34 ± 3 | 28 ± 2 | 28 ± 3 | 11 ± 3 | 16 ± 2 |
| 11-HETE | 127 ± 17 | 164 ± 28 | 138 ± 15 | 119 ± 14 | 70 ± 24 | 73 ± 21 |
| PGD_2_ | 311 ± 151 | 204 ± 74 | 96 ± 26 | 161 ± 113 | 107 ± 49 | 217 ± 145 |
| PGE_2_ | 100 ± 25 | 164 ± 79 | 75 ± 24 | 43 ± 5 | 41 ± 4 | 61 ± 13 |
| 15-keto PGE_2_ | 12 ± 5 | 23 ± 8 | 6 ± 1 | 4 ± 2 | 10 ± 3 | 27 ± 21 |
| PGF_2_α | 71 ± 10 | 75 ± 16 | 36 ± 12 | 43 ± 6 | 66 ± 7 | 92 ± 34 |
| TXB_2_ | 652 ± 39 | 1319 ± 579 | 820 ± 127 | 633 ± 106 | 727 ± 158 | 1087 ± 314 |
| PGE_1_ | 18 ± 6 | 19 ± 9 | 9 ± 4 | 11 ± 5 | 18 ± 8 | 10 ± 4 |
| AA | 1551711  ± 215253 | 2599190  ± 826482 | 802323  ± 205952 | 574966  ± 208690 | 389917  ± 137742 | 492721  ± 187681 |
| EPA | 45801  ± 9587 | 71141  ± 11658 | 19861  ± 6118 | 11927  ± 3656 | 9861  ± 4822 | 13055  ± 5445 |
| DHA | 952579  ± 136452 | 1398830  ± 313777 | 523032  ± 205097 | 332727  ± 105930 | 259136  ± 106023 | 310940  ± 150082 |
| DPA | 32156  ± 6485 | 46131  ± 13909 | 14492  ± 5293 | 9597  ± 2937 | 7480  ± 3511 | 8205  ± 4654 |

Data are shown as mean ± s.e.m; pg/0.2 mg protein. SACM: *S. aureus*-conditioned medium; WT: wildtype; KO: *Agmo*-deficient.

**Table. S6 Lipid mediator profile in Ca^2+^-ionophore A23187-stimulated BMDMs from male mice.**

|  | M0_WT | M0_KO | M1_WT | M1_KO | M2_WT | M2_KO |
| --- | --- | --- | --- | --- | --- | --- |
| 17-HDHA | 38 ± 28 | 52 ± 15 | 52 ± 24 | 185 ± 118 | 12 ± 8 | 21 ± 11 |
| 14-HDHA | 5 ± 4 | 13 ± 4 | 11 ± 5 | 33 ± 26 | 3 ± 2 | 5 ± 2 |
| 17-HDPA | 36 ± 20 | 72 ± 16 | 69 ± 22 | 150 ± 66 | 17 ± 8 | 24 ± 8 |
| 14-HDPA | 19 ± 13 | 52 ± 17 | 33 ± 10 | 58 ± 27 | 6 ± 3 | 5 ± 3 |
| 15-HEPE | 26 ± 11 | 30 ± 3 | 34 ± 8 | 63 ± 20 | 10 ± 6 | 24 ± 9 |
| 12-HEPE | 12 ± 5 | 15 ± 2 | 16 ± 3 | 17 ± 5 | 7 ± 3 | 8 ± 3 |
| 15-HETE | 79 ± 45 | 133 ± 29 | 178 ± 50 | 586 ± 281 | 90 ± 49 | 143 ± 43 |
| 12-HETE | 16 ± 8 | 18 ± 2 | 27 ± 7 | 63 ± 39 | 12 ± 4 | 14 ± 6 |
| 5-HEPE | 72 ± 28 | 87 ± 10 | 114 ± 34 | 102 ± 11 | 40 ± 22 | 43 ± 12 |
| 5-HETE | 41 ± 18 | 75 ± 15 | 120 ± 37 | 175 ± 94 | 38 ± 18 | 40 ± 15 |
| LTB_4_ isomers | 2 ± 1 | 8 ± 3 | 0 ± 0 | 8 ± 7 | 4 ± 2 | 7 ± 4 |
| LTB_4_ | 4 ± 2 | 5 ± 2 | 1 ± 1 | 4 ± 2 | 4 ± 3 | 5 ± 2 |
| 7-HDHA | 9 ± 4 | 12 ± 2 | 14 ± 4 | 12 ± 1 | 6 ± 2 | 7 ± 2 |
| 4-HDHA | 6 ± 3 | 18 ± 5 | 17 ± 5 | 25 ± 11 | 5 ± 3 | 7 ± 2 |
| 7-HDPA | 20 ± 10 | 59 ± 14 | 61 ± 19 | 88 ± 20 | 15 ± 9 | 18 ± 4 |
| 18-HEPE | 146 ± 45 | 150 ± 14 | 133 ± 40 | 146 ± 33 | 128 ± 83 | 175 ± 66 |
| 5,15-diHETE | 40 ± 7 | 52 ± 12 | 76 ± 21 | 110 ± 21 | 57 ± 22 | 62 ± 17 |
| 11-HEPE | 21 ± 10 | 29 ± 3 | 50 ± 12 | 94 ± 23 | 17 ± 12 | 31 ± 9 |
| 11-HETE | 36 ± 19 | 81 ± 16 | 234 ± 100 | 677 ± 276 | 65 ± 45 | 128 ± 39 |
| PGD_2_ | 43 ± 21 | 151 ± 35 | 57 ± 12 | 126 ± 38 | 74 ± 46 | 139 ± 34 |
| PGE_2_ | 27 ± 13 | 76 ± 10 | 179 ± 66 | 394 ± 115 | 56 ± 34 | 83 ± 19 |
| 15-ketoPGE_2_ | 5 ± 2 | 12 ± 1 | 12 ± 3 | 19 ± 4 | 8 ± 4 | 11 ± 1 |
| PGF_2_α | 24 ± 9 | 42 ± 7 | 75 ± 17 | 127 ± 30 | 47 ± 23 | 55 ± 11 |
| TXB_2_ | 115 ± 55 | 334 ± 52 | 946 ± 273 | 2710 ± 852 | 533 ± 351 | 1021 ± 264 |
| PGE_1_ | 2 ± 0 | 4 ± 1 | 13 ± 5 | 21 ± 7 | 4 ± 2 | 4 ± 1 |
| AA | 141114  ± 80544 | 323013  ± 78688 | 269253  ± 122090 | 633035  ± 276597 | 216157  ± 136890 | 278303  ± 105484 |
| EPA | 6169  ± 3781 | 11323  ± 2525 | 8478  ± 4225 | 15285  ± 6068 | 5412  ± 3908 | 6735  ± 2653 |
| DHA | 71544  ± 39617 | 163815  ± 35019 | 171335  ± 85066 | 286611  ± 126445 | 74273  ± 45191 | 66684  ± 23418 |
| DPA | 2159  ± 1305 | 4511  ± 1116 | 3769  ± 1705 | 7648  ± 3353 | 1895  ± 1190 | 1736  ± 525 |

Data are shown as mean ± s.e.m; pg/0.2 mg protein. WT: wildtype; KO: *Agmo*-deficient.

**Table. S7 Lipid mediator profile in non-infected and Salmonella-infected adipose tissues from female and male mice.**

| female | vWAT | | sWAT | | BAT | |
| --- | --- | --- | --- | --- | --- | --- |
| non-infected | WT | KO | WT | KO | WT | KO |
| PGD_2_ | 17 ± 3 | 23 ± 7 | 81 ± 33 | 113 ± 44 | 28 ± 9 | 18 ± 1 |
| PGE_2_ | 30 ± 6 | 75 ± 28 | 158 ± 47 | 177 ± 34 | 38 ± 12 | 37 ± 10 |
| PGF_2_α | 24 ± 8 | 46 ± 17 | 67 ± 22 | 65 ± 12 | 40 ± 15 | 30 ± 7 |
| TXB_2_ | 11 ± 3 | 20 ± 9 | 44 ± 13 | 54 ± 12 | 57 ± 17 | 47 ± 12 |
| AA | 784514  ± 192275 | 935104  ± 255130 | 796954  ± 169054 | 1302192  ± 345234 | 924249  ± 181384 | 902109  ± 127900 |
| male | vWAT | | sWAT | | BAT | |
| non-infected | WT | KO | WT | KO | WT | KO |
| PGD_2_ | 18 ± 4 | 17 ± 4 | 58 ± 11 | 107 ± 43 | 17 ± 5 | 13 ± 2 |
| PGE_2_ | 26 ± 5 | 31 ± 8 | 180 ± 43 | 324 ± 64 | 41 ± 12 | 37 ± 11 |
| PGF_2_α | 21 ± 3 | 20 ± 4 | 80 ± 21 | 114 ± 30 | 31 ± 9 | 30 ± 8 |
| TXB_2_ | 15 ± 5 | 10 ± 1 | 50 ± 8 | 75 ± 19 | 61 ± 15 | 22 ± 3 |
| AA | 668897  ± 98746 | 725907  ± 116590 | 855355  ± 177010 | 957583  ± 175372 | 969050  ± 239352 | 953792  ± 86903 |
| female | vWAT | | sWAT | | BAT | |
| infected | WT | KO | WT | KO | WT | KO |
| PGD_2_ | 17 ± 3 | 19 ± 1 | 143 ± 33 | 149 ± 46 | 31 ± 2 | 24 ± 3 |
| PGE_2_ | 50 ± 13 | 51 ± 9 | 92 ± 4 | 106 ± 42 | 115 ± 20 | 100 ± 28 |
| PGF_2_α | 45 ± 13 | 49 ± 13 | 57 ± 7 | 83 ± 40 | 94 ± 17 | 65 ± 10 |
| TXB_2_ | 35 ± 9 | 38 ± 9 | 87 ± 18 | 89 ± 32 | 55 ± 14 | 64 ± 9 |
| AA | 1223442  ± 39761 | 1400104  ± 94068 | 1367188  ± 97377 | 1673930  ± 57949 | 2054125  ± 144611 | 2479902  ± 271540 |
| male | vWAT | | sWAT | | BAT | |
| infected | WT | KO | WT | KO | WT | KO |
| PGD_2_ | 28 ± 5 | 21 ± 5 | 145 ± 34 | 90 ± 29 | 37 ± 7 | 23 ± 2 |
| PGE_2_ | 52 ± 7 | 38 ± 7 | 232 ± 19 | 204 ± 41 | 110 ± 20 | 88 ± 25 |
| PGF_2_α | 40 ± 8 | 32 ± 13 | 109 ± 12 | 116 ± 16 | 72 ± 11 | 49 ± 5 |
| TXB_2_ | 21 ± 4 | 24 ± 8 | 125 ± 18 | 119 ± 27 | 57 ± 14 | 32 ± 2 |
| AA | 1168207  ± 147841 | 1209828  ± 195949 | 1086672  ± 130968 | 1312434  ± 128426 | 2344225  ± 274297 | 1792672  ± 115719 |

Data are shown as mean ± s.e.m; pg/mg tissue. WT: wildtype; KO: *Agmo*-deficient; vWAT: visceral white adipose tissue; sWAT: subcutaneous white adipose tissue; BAT: brown adipose tissue.
